# A general kernel machine regression framework using principal component analysis for jointly testing main and interaction effects: Applications to human microbiome studies

**DOI:** 10.1093/nargab/lqae148

**Published:** 2024-11-12

**Authors:** Hyunwook Koh

**Affiliations:** Department of Applied Mathematics and Statistics, The State University of New York, Korea, Incheon 21985, South Korea

## Abstract

The effect of a treatment on a health or disease response can be modified by genetic or microbial variants. It is the matter of interaction effects between genetic or microbial variants and a treatment. To powerfully discover genetic or microbial biomarkers, it is crucial to incorporate such interaction effects in addition to the main effects. However, in the context of kernel machine regression analysis of its kind, existing methods cannot be utilized in a situation, where a kernel is available but its underlying real variants are unknown. To address such limitations, I introduce a general kernel machine regression framework using principal component analysis for jointly testing main and interaction effects. It begins with extracting principal components from an input kernel through the singular value decomposition. Then, it employs the principal components as surrogate variants to construct three endogenous kernels for the main effects, interaction effects, and both of them, respectively. Hence, it works with a kernel as an input without knowing its underlying real variants, and also detects either the main effects, interaction effects, or both of them robustly. I also introduce its omnibus testing extension to multiple input kernels, named OmniK. I demonstrate its use for human microbiome studies.

## Introduction

In this study, I pay attention to a randomized clinical trial or an observational study, where the effect of a treatment (e.g. medical treatment, environmental factor, health policy) on a health or disease response can be modified by genetic or microbial variants. The effect of a treatment can vary by subjects because their genome or metagenome can boost it or lower it. It surely is the matter of interaction effects between genetic or microbial variants and a treatment, which is also a key consideration in personalized medicine and precision health approaches (1). I note especially that to powerfully discover genetic or microbial biomarkers, it is crucial to incorporate such interaction effects in addition to the main effects (2–4). That is, genetic or microbial biomarkers can be better identified based on whether they influence a health or disease response either (i) directly through main effects, (ii) indirectly through interaction effects or (iii) both directly and indirectly through main and interaction effects. We can enhance the test power by jointly testing main and interaction effects (2–4).

A kernel machine regression analysis has been widely utilized in human genome (5,6) or metagenome (7) studies to test the relationship between genetic or microbial variants and a health or disease response adjusting for covariates (e.g. age, sex). This approach offers significant advantages: (i) enhanced test power through efficient multifactorial aggregation, allowing for the simultaneous consideration of multiple genetic or microbial factors and (ii) broad applicability through flexible regression-based modeling, accommodating a wide range of study designs and data types (5–16). However, in the context of kernel machine regression analysis for jointly testing main and interaction effects, existing methods have limitations as described below.

Two notable kernel machine regression methods of its kind are composite kernel likelihood ratio test (CKLRT) (17) and composite kernel association test (CKAT) (18). CKLRT (17) and CKAT (18) construct a composite kernel as a weighted average of the two kernels that correspond to the main and interaction effects, respectively, and then optimize their weights using the minimum *P*-value approach (19) in significance testing. However, they cannot work with a kernel as an input without knowing its underlying real variants. That is, to construct a kernel for the interaction effects, its underlying real variants are needed because the interaction effects are modeled based on the product terms between variants and a treatment. For the traditional linear kernel, for example, it is easy to retrieve its underlying real variants: they are simply the original variants. However, a kernel is referred in general as any positive semi-definite matrix (20) that reflects pairwise (i.e. subject-by-subject) similarities (or relatedness), which enables us to capture possibly complex patterns of the relationships between variants and a response. It is not always easy to retrieve its underlying real variants. Relevant examples are ecological kernels (21–25) that are widely utilized in human microbiome studies. To brief, the human microbiome is the entire ecosystem of all microbes living in the human body, and the ecological kernels reflect pairwise (i.e. host-by-host) similarities (or relatedness) in microbiome composition. The ecological kernels (21–25) are defined based on various ingredients, such as microbial presence-absence or abundance information and non-phylogenetic or phylogenetic information, and they have advanced our understanding of microbial ecology leading to enhanced test power in significance testing eventually (7,13–15). However, their underlying real variants are hard to retrieve because of the high complexity in their mathematical definitions. Therefore, CKLRT (17) and CKAT (18) cannot work with an ecological kernel as an input, for which its underlying real variants are unknown.

In this paper, I introduce a general kernel machine regression framework using principal component analysis for jointly testing main and interaction effects. It begins with extracting principal components from an input kernel (e.g. ecological kernel) through the singular value decomposition. Then, it employs the principal components as surrogate variants for the underlying real variants to construct three endogenous kernels for the (i) main effects, (ii) interaction effects and (iii) both of the main and interaction effects, respectively. Hence, it works with a kernel as an input without knowing its underlying real variants. It also detects either the main effects, interaction effects, or both of them robustly through omnibus testing across the three endogenous kernels. Note also that there are diverse input kernels in reality, and they return all different results (7). For this reason, omnibus testing has recently been widely utilized for unified and powerful testing across multiple input kernels. Of course, CKLRT (17) and CKAT (18) conduct omnibus testing to optimize their weights between the two endogenous kernels for the main and interaction effects as described above; yet, they process multiple input kernels, such as the linear, quadratic and identity-by-descent kernels, individually, possibly leading to inflated family-wise test error rates. Therefore, I also introduce an omnibus testing extension to multiple input kernels (e.g. ecological kernels), named as OmniK. While its methodology can apply to various disciplines, I demonstrate it utilizing ecological kernels (21–25) in human microbiome studies. I also demonstrate its outperformance in significance testing, compared with other existing methods, through extensive simulation experiments. I apply it to two real microbiome datasets on (i) the gut microbiome and its interaction with a diet method on body weight for rhesus monkeys (26); and (ii) the oral microbiome and its interaction with e-cigarette smoking on gingival inflammation (27). Finally, I summarize all and finish with concluding remarks.

## Materials and methods

### Notations and Models

Suppose that there are *n* subjects (*i* = 1, …, *n*), *p* variants (e.g., genetic or microbial variants) (*j* = 1, …, *p*), a treatment (e.g. medical treatment, environmental factor, health policy), and *q* covariates (e.g. age, sex) (*k* = 1, …, *q*) possibly with the high-dimensionality of *p* ≫ *n*. Then, let *y* denote an *n* × 1 vector for a response (e.g. health or disease status), *X* denote an *n* × *p* matrix for variants, *T* denote an *n* × 1 vector for a treatment, and *Z* denote an *n* × *q* matrix for covariates. Then, for a continuous response, I consider a linear regression model as in Eq. (1),


(1) \begin{equation*} y = \alpha _1 + T \alpha _2 + Z \alpha _3 + f(X) \beta _1 + (f(X) \odot T) \beta _2 + \epsilon , \end{equation*}

where *f*(·) is a basis function for a linear or non-linear transformation; *f*(*X*)⊙*T* is an *n* × *p* matrix for the Hadamard products (i.e. interaction terms) between *f*(*X*) and *T*; *α*_1_ is a scalar for the intercept; *α*_2_ is a scalar for the main effect of a treatment; *α*_3_ is a *q* × 1 vector for the main effects of covariates: *α*_3_ = (*α*_31_, …, *α*_3*q*_)^*T*^: *β*_1_ is a *p* × 1 vector for the main effects of variants: *β*_1_ = (*β*_11_, …, *β*_1*p*_)^*T*^; *β*_2_ is a *p* × 1 vector for the interaction effects between variants and a treatment: *β*_2_ = (*β*_21_, …, *β*_2*p*_)^*T*^; and *ε* is an *n* × 1 vector for the independently and identically distributed errors with mean zero and variance *σ*^2^: *ε* = (*ε*_1_, …, *ε*_*n*_)^*T*^. Likewise, for a binary response, I consider a logistic regression model as in Eq. (2),


(2) \begin{eqnarray*} \text{logit}(E(y|M, T, Z)) &=& \alpha _1 + T \alpha _2 + Z \alpha _3 + f(X) \beta _1 \nonumber \\ && \, + (f(X) \odot T) \beta _2. \end{eqnarray*}

For significance testing, we are interested in testing the null hypothesis of no main and interaction effects with its alternative hypothesis to be of three sub-statements on the presence of main effects, the presence of interaction effects, or the presence of both main and interaction effects as in Eq. (3).


\begin{eqnarray*} H_0: \beta _1 &=& (\beta _{11}, \dots , \beta _{1p})^T = 0_{p \times 1} \text{ & } \beta _2 \\ &=& (\beta _{21}, \dots , \beta _{2p})^T = 0_{p \times 1} \end{eqnarray*}

\begin{eqnarray*} \Longleftrightarrow H_0: \beta _1^T \beta _1 = 0 \text{ & } \beta _2^T \beta _2 = 0 \end{eqnarray*}

\begin{eqnarray*} vs. \end{eqnarray*}

(3) \begin{equation*} H_1: \beta _1^T \beta _1 \ne 0, \beta _2^T \beta _2 \ne 0, \text{ or } \beta _1^T \beta _1 \ne 0 \text{ & } \beta _2^T \beta _2 \ne 0 \end{equation*}

Here, a treatment *T* can be independent to variants *X* and covariates *Z* as in a randomized clinical trial, where a treatment assignment is made randomly; or *T* can be dependent to variants *X* and covariates *Z* as in an observational study. Note that if *f*(·) is the identity (or linear) transformation, the original variants *X* are linearly related to a response. Though *f*(·) can be any linear or non-linear transformation that is also known or unknown in general.

### General kernel machine regression framework

A kernel is referred in general as any *n* × *n* positive semi-definite matrix (20) that reflects pairwise (i.e. subject-by-subject) similarities (or relatedness). There are also diverse ways to measure the similarities, and their formulas are not always simple as for the linear kernel; that is, a kernel does not always need to be a gram matrix of the original variant matrix *XX*^*T*^. Nonetheless, a kernel should be a gram matrix of a linearly or non-linearly transformed variant matrix *f*(*X*)*f*(*X*)^*T*^, though *f*(·) is possibly unknown. Furthermore, since there are diverse kernels, there are indeed multiple possibly unknown linearly or non-linearly transformed variant matrices: *f*_(*h*)_(*X*)*f*_(*h*)_(*X*)^*T*^, where *h* is an index for a kernel among diverse kernels, *h* ∈ Γ. In a later section of *Application Note: Human Microbiome and Ecological Kernels*, I describe such a situation using ecological kernels in human microbiome studies. In theory, a kernel can also be constructed without any original variant matrix *X* (20); hence, *X* can be absent even in our imagination.

However, it is interesting to note the Mercer’s theorem (20) that even when *f*_(*h*)_(*X*) is unknown, we can obtain its orthogonal lower-dimensional linear representations, referred as surrogate variants in this paper, through the singular value decomposition of a kernel as in Eq. (4).


(4) \begin{eqnarray*} K_{(h)} &=& f_{(h)}(X)f_{(h)}(X)^T = U_{(h)}D_{(h)}D_{(h)}^TU_{(h)}^T \nonumber \\ &=& U_{(h)}D_{(h)}^2U_{(h)}^T = X_{(h)}^* X_{(h)}^{*T}. \end{eqnarray*}

where *h* is an index for a kernel among diverse input kernels, *h* ∈ Γ; *K*_(*h*)_ is an *n* × *n* positive semi-definite kernel; *U*_(*h*)_ is an *n* × *n* matrix for left singular vectors; and *D*_(*h*)_ is an *n* × *n* diagonal matrix for ordered non-negative singular values (i.e. λ_(*h*)1_ ≥ … ≥ λ_(*h*)*n*_). Here, we can remove the last few monotone principal components that make their singular values to be exactly zero from consideration (20), resulting in $X_{(h)}^*$ to be an *n* × *df* full degrees-of-freedom principal component matrix with *df* ≤ *n*. Then, the columns of $X_{(h)}^*$ are *df* principal components that are the orthogonal lower-dimensional linear representations of the unknown *p* transformed variants, that is, the columns of *f*_(*h*)_(*X*), where *f*_(*h*)_(·) is unknown.

I reformulate the models using the *df* principal components as surrogate variants of the unknown *p* transformed variants as in Eq. (5) for Eq. (1) and as in Eq. (6) for Eq. (2).


(5) \begin{eqnarray*} y = \alpha _1^* + T \alpha _2^* + Z \alpha _3^* + X_{(h)}^* \beta _1^* + (X_{(h)}^* \odot T) \beta _2^* + \epsilon , \end{eqnarray*}

and


(6) \begin{eqnarray*} \text{logit}(E(y|X, T, Z)) &=& \alpha _1^* + T \alpha _2^* + Z \alpha _3^* + X_{(h)}^* \beta _1^* \nonumber \\ &&+\, (X_{(h)}^* \odot T) \beta _2^*. \end{eqnarray*}

where $X_{(h)}^*$ is an *n* × *df* matrix for the surrogate variants; $X_{(h)}^* \odot T$ is an *n* × *df* matrix for the Hadamard products (i.e. interaction terms) between $X_{(h)}^*$ and *T*; $\alpha _1^*$ is a scalar for the intercept; $\alpha _2^*$ is a scalar for the main effect of a treatment; $\alpha _3^*$ is a *q* × 1 vector for the main effects of covariates: $\alpha _3^* = (\alpha _{31}^*, \dots , \alpha _{3q}^*)^T$: $\beta _1^*$ is a *df* × 1 vector for the main effects of surrogate variants: $\beta _1^* = (\beta _{11}^*, \dots , \beta _{1df}^*)^T$; $\beta _2^*$ is a *df* × 1 vector for the interaction effects between surrogate variants and a treatment: $\beta _2^* = (\beta _{21}^*, \dots , \beta _{2p}^*)^T$; and *ε* is an *n* × 1 vector for the independently and identically distributed errors with mean zero and variance *σ*^2^: *ε* = (*ε*_1_, …, *ε*_*n*_)^*T*^. The model specifications between Eq. (1) and Eq. (5) or between Eq. (2) and Eq. (6) are different; though their null models are identical to Eq. (7) and Eq. (8), resulting in the same null hypothesis of Eq. (3) to be tested.


(7) \begin{eqnarray*} y = \alpha _{10} + T \alpha _{20} + Z \alpha _{30} + \epsilon _0 \end{eqnarray*}

and


(8) \begin{eqnarray*} \text{logit}(E(y|T, Z)) = \alpha _{10} + T \alpha _{20} + Z \alpha _{30}. \end{eqnarray*}

Note that the benefit of Eq. (5) and Eq. (6) are in their capability to survey the interaction effects including the product terms of $X_{(h)}^* \odot T$ even when *f*_(*h*)_(*X*) is unknown. We can also flexibly survey any linear or non-linear relationships depending on which input kernel *h* is used.

I formulate its variance component score test statistic for each of the main effects ($T_{(h)}^M$), interaction effects ($T_{(h)}^I$) and both of them ($T_{(h)}^{B}$) as in Eq. (9).


(9) \begin{eqnarray*} T_{(h)}^M &=& (y - \hat{y}_0)^T K_{(h)}^M (y - \hat{y}_0), T_{(h)}^I = (y - \hat{y}_0)^T K_{(h)}^I (y - \hat{y}_0), \nonumber \\ \text{ and } T_{(h)}^{B} &=& (y - \hat{y}_0)^T K_{(h)}^{B} (y - \hat{y}_0). \end{eqnarray*}

Here, the kernels, $K_{(h)}^M$, $K_{(h)}^I$ and $K_{(h)}^{B}$, are three endogenous *n* × *n* positive semi-definite gram matrices of $X_{(h)}^*$, $(X_{(h)}^* \odot T)$ and $(X_{(h)}^* | X_{(h)}^* \odot T)$, respectively. That is, $K_{(h)}^M = X_{(h)}^*X_{(h)}^{*T}$, $K_{(h)}^I = (X_{(h)}^* \odot T)(X_{(h)}^* \odot T)^T$, and $K_{(h)}^{B} = (X_{(h)}^* | X_{(h)}^* \odot T)(X_{(h)}^* | X_{(h)}^* \odot T)^T$. The residual vector $y - \hat{y}_0$ reflects the remaining responses regressing out the main effects of a treatment and covariates based on the null models of Eqs. (7) and (8). Then, $T_{(h)}^M$, $T_{(h)}^I$ and $T_{(h)}^{B}$ are estimates on (i) the sum of squared main effects $\hat{\beta }_1^{*T} \hat{\beta }_1^*$ assuming no interaction effects, (ii) the sum of squared interaction effects $\hat{\beta }_2^{*T} \hat{\beta }_2^*$ assuming no main effects, and (iii) the sum of squared main and interaction effects $\hat{\beta }_1^{*T} \hat{\beta }_1^* + \hat{\beta }_2^{*T} \hat{\beta }_2^*$, respectively. Therefore, we can expect that $K_{(h)}^{M}$ is powerful when only the main effects exist; $K_{(h)}^{I}$ is powerful when only the interaction effects exist; and $K_{(h)}^{B}$ is powerful when both of the main and interaction effects exist.

To obtain a null test statistic value on the absence of main and interaction effects, we can rearrange the residual vector $y - \hat{y}_0$ for either of $T_{(h)}^M$, $T_{(h)}^I$ or $T_{(h)}^{B}$. Then, we can calculate a *P*-value as the proportion of null test statistic values larger than or equal to the observed test statistic value as in Eq. (10).


(10) \begin{eqnarray*} P_{(h)}^{M} &=& \frac{1}{R} \sum _{r = 1}^{R} I(T_{(h)}^{M(r)} \ge T_{(h)}^{M(obs)}), \nonumber \\ P_{(h)}^{I} &=& \frac{1}{R} \sum _{r = 1}^{R} I(T_{(h)}^{I(r)} \ge T_{(h)}^{I(obs)}), \nonumber \\ \text{ and } P_{(h)}^{B} &=& \frac{1}{R} \sum _{r = 1}^{R} I(T_{(h)}^{B(r)} \ge T_{(h)}^{B(obs)}), \end{eqnarray*}

where *R* is the number of rearrangements with *r* = 1, …, *R* and *I*(·) is an indicator function.

However, if we perform these tests individually, the family-wise test error rate can be inflated. Hence, I use the minimum *P*-value approach (19) for omnibus testing. The minimum *P*-value approach (19) employs the minimum *P*-value among the *P*-values from multiple tests as a test statistic as in Eq. (11).


(11) \begin{eqnarray*} T_{(h)} = \min \lbrace P_{(h)}^{M}, P_{(h)}^{I}, P_{(h)}^{B}\rbrace . \end{eqnarray*}

Its rationale is to reflect the strongest evidence of significance, which enables to approach the highest test power across $T_{(h)}^M$, $T_{(h)}^I$ and $T_{(h)}^{B}$. Hence, *T*_(*h*)_ can robustly detect either the main effects, interaction effects, or both of them.

Since the minimum *P*-value itself is a test statistic, we need its null distribution to calculate a *P*-value to report as a final result. To obtain a null test statistic value, we can rearrange the residual vector $y - \hat{y}_0$ for all of $T_{(h)}^M$, $T_{(h)}^I$ and $T_{(h)}^{B}$, simultaneously, as in Eq. (12).


\begin{eqnarray*} P_{(h)(r^{\prime })}^{M} &=& \frac{1}{R - 1} \sum _{r^{\prime } \ne r} I(T_{(h)}^{M(r)} \ge T_{(h)}^{M(r^{\prime })}), \nonumber \\ P_{(h)(r^{\prime })}^{I} &=&\frac{1}{R - 1} \sum _{r^{\prime } \ne r} I(T_{(h)}^{I(r)} \ge T_{(h)}^{I(r^{\prime })}), \end{eqnarray*}

(12) \begin{eqnarray*} P_{(h)(r^{\prime })}^{B} &=& \frac{1}{R - 1} \sum _{r^{\prime } \ne r} I(T_{(h)}^{B(r)} \ge T_{(h)}^{B(r^{\prime })}), \nonumber \\ \text{ and } T_{(h)(r^{\prime })} &=& \min \lbrace P_{(h)(r^{\prime })}^{M}, P_{(h)(r^{\prime })}^{I}, P_{(h)(r^{\prime })}^{B}\rbrace . \end{eqnarray*}

where *R* is the number of rearrangements with *r* = 1, …, *R* and *r*′ = 1, …, *R*. Then, we can calculate a *P*-value as the proportion of null test statistic values smaller than or equal to the observed test statistic value as in Eq. (13).


(13) \begin{eqnarray*} P_{(h)} = \frac{1}{R} \sum _{r^{\prime } = 1}^{R} I(T_{(h)(r^{\prime })} \le T_{(h)(obs)}). \end{eqnarray*}

In this section, I described a general kernel machine regression framework for each input kernel (i.e. each *h* in Γ) whether its underlying real variants are known or unknown. In a later section of *Omnibus Testing on Multiple Input Kernels: OmniK*, I describe its omnibus testing extension to multiple input kernels, which is for a unified and powerful test across all *h*’s in Γ.

### Application note: human microbiome and ecological kernels

The human microbiome field is rapidly growing due to the recent advancement in next-generation sequencing that enables more accurate microbiome profiling at a dramatically lowered cost (28). While there are diverse research directions, here I survey if the microbiome (*X*) influences a human health or disease response (*y*) either directly or indirectly through its interaction with a treatment (*T*) adjusting for the main effects of a treatment (*T*) and covariates (*Z*) through kernel machine regression analysis. For this, we need a kernel *K*_(*h*)_ as an input in Eq. (4), though *f*_(*h*)_(*X*) is not necessary and can be unknown. Then, we can simply follow all the remaining analytic procedures. As in (1,7), I define a kernel transforming an ecological distance or dissimilarity (also known as β-diversity) matrix through Eq. (14).


(14) \begin{eqnarray*} {K_{(h)} = - \frac{1}{2} (I - \frac{11^{\prime }}{n}) \Delta _{(h)}^2 (I - \frac{11^{\prime }}{n}),} \end{eqnarray*}

where *h* is an index among diverse ecological distance or dissimilarity measures, *h* ∈ Γ; $\Delta _{(h)}^2$ is an *n* × *n* matrix for element-wise squared ecological distances or dissimilarities; *I* is an *n* × *n* identity matrix; and 1 is an *n* × 1 vector of 1s. Here, Δ_(*h*)_ reflects pairwise (i.e., subject-by-subject) distances or dissimilarities in microbiome composition, while *K*_(*h*)_ reflects their similarities; hence, Eq. (14) is a formula to convert distance or dissimilarity reversely to similarity.

The most widely used ecological distance or dissimilarity measures are Jaccard distance (21), Bray-Curtis dissimilarity (22), unweighted UniFrac distance (23), generalized UniFrac distance (24) and weighted UniFrac distance (25). Accordingly, we can call their ecological kernels as Jaccard (21), Bray-Curtis (22), unweighted UniFrac (23), generalized UniFrac (24) and weighted UniFrac (25) kernels. These ecological kernels are suitable to different patterns of the relationships (7). That is, presence-absence kernels (e.g. Jaccard (21) and unweighted UniFrac (23)) are powerful when rare variants are related to a response, while abundance-based kernels (e.g. Bray-Curtis (22), unweighted UniFrac (23), generalized UniFrac (24) and weighted UniFrac (25)) are powerful when common variants are related to a response. This is because common variants tend to be present across all subjects; hence, it is hard to detect their relationships using presence-absence information. On the contrary, presence-absence information can be useful to detect discrete non-linear patterns of the relationships for rare variants. Moreover, phylogenetic kernels (e.g. unweighted UniFrac (23), generalized UniFrac (24) and weighted UniFrac (25)) are powerful when phylogenetically close variants are related to a response, while non-phylogenetic kernels (e.g. Jaccard (21) and Bray-Curtis (22)) are powerful when non-phylogenetic variants are related to a response. Interestingly, the generalized UniFrac kernel (24) is of a tuning parameter (0 ≤ α ≤ 1) to adjust the degree of abundance information to be reflected; as such, the generalized UniFrac kernel resembles the unweighted UniFrac kernel (23) when α is small, while it resembles the weighted UniFrac kernel (25) when α is large.

Note that the *p* real variants that underlie each ecological kernel, *f*_(*h*)_(*X*), *h* ∈ Γ, are hard to retrieve because *f*_(*h*)_(·) is unknown due to the complex mathematical operations to define an ecological distance or dissimilarity (e.g., presence-absence vs. abundance, non-phylogenetic versus phylogenetic) and its conversion to an ecological kernel. However, importantly, through the singular value decomposition of a kernel as in Eq. (4), we can obtain their surrogate variants (i.e. orthogonal lower-dimensional linear representations). This enables us to conduct kernel machine regression analysis with an ecological kernel as an input without knowing its underlying *p* real variants.

### Omnibus testing on multiple input kernels: OmniK

The performance of kernel machine regression analysis can vary depending on which input kernel (e.g. ecological kernel) is used (5–7). Hence, I introduce OmniK for a unified and powerful test across multiple input kernels. Below is its methodological description.

I first formulate its test statistics for each endogenous kernel on the main effects, interaction effects or both of them as in Eq. (15).


(15) \begin{eqnarray*} T_{OmniK}^M = \min _{h \in \Gamma } P_{(h)}^{M}, T_{OmniK}^I = \min _{h \in \Gamma } P_{(h)}^{I}, \text{ and } T_{OmniK}^B = \min _{h \in \Gamma } P_{(h)}^{B}, \nonumber \\ \end{eqnarray*}

where *h* is an index for a kernel among diverse input kernels, *h* ∈ Γ; and $P_{(h)}^{M}$, $P_{(h)}^{I}$ and $P_{(h)}^{B}$ are the *P*-values for each endogenous kernel given *h* as in Eq. (10). Here, we can see that $T_{OmniK}^M$, $T_{OmniK}^I$ and $T_{OmniK}^B$ are the minimum *P*-values across the multiple input kernels (i.e. *h*’s in Γ) for each endogenous kernel on the main effects, interaction effects or both of them. Then, they reflect the strongest evidence of significance, which enables to approach the highest test power across the multiple input kernels (i.e. *h*’s in Γ), for the main effects, interaction effects or both of them.

To obtain a null test statistic value, we can rearrange the residual vector $y - \hat{y}_0$ for all of $T_{(h)}^M$’s, $T_{(h)}^I$’s and $T_{(h)}^{B}$’s in Γ, simultaneously, as in Eq. (16).


\begin{eqnarray*} T_{OmniK(r^{\prime })}^M &=& \min _{h \in \Gamma } \lbrace \frac{1}{R - 1} \sum _{r^{\prime } \ne r} I(T_{(h)}^{M(r)} \ge T_{(h)}^{M(r^{\prime })})\rbrace , \nonumber \\ T_{OmniK(r^{\prime })}^I &=& \min _{h \in \Gamma } \lbrace \frac{1}{R - 1} \sum _{r^{\prime } \ne r} I(T_{(h)}^{I(r)} \ge T_{(h)}^{I(r^{\prime })})\rbrace , \end{eqnarray*}

(16) \begin{eqnarray*} \text{and }\ T_{OmniK(r^{\prime })}^B = \min _{h \in \Gamma } \lbrace \frac{1}{R - 1} \sum _{r^{\prime } \ne r} I(T_{(h)}^{B(r)} \ge T_{(h)}^{B(r^{\prime })})\rbrace , \end{eqnarray*}

where *R* is the number of rearrangements with *r* = 1, …, *R* and *r*′ = 1, …, *R*. Then, we can calculate a *P*-value for each of $T_{OmniK}^M$, $T_{OmniK}^I$ and $T_{OmniK}^B$ as the proportion of null test statistic values smaller than or equal to the observed test statistic value as in Eq. (17).


\begin{eqnarray*} P_{OmniK}^M &=& \frac{1}{R} \sum _{r^{\prime } = 1}^{R} I(T_{OmniK(r^{\prime })}^M \le T_{OmniK(obs)}^M), \nonumber \\ P_{OmniK}^I &=& \frac{1}{R} \sum _{r^{\prime } = 1}^{R} I(T_{OmniK(r^{\prime })}^I \le T_{OmniK(obs)}^I), \end{eqnarray*}

(17) \begin{eqnarray*} \text{and }\ P_{OmniK}^B = \frac{1}{R} \sum _{r^{\prime } = 1}^{R} I(T_{OmniK(r^{\prime })}^B \le T_{OmniK(obs)}^B). \end{eqnarray*}

However, again, if we perform these tests individually, the family-wise test error rate can still be inflated. Therefore, finally, I formulate the test statistic for OmniK as in Eq. (18).


(18) \begin{eqnarray*} T_{OmniK} = \min \lbrace P_{OmniK}^{M}, P_{OmniK}^{I}, P_{OmniK}^{B}\rbrace . \end{eqnarray*}

To obtain a null test statistic value, we can rearrange the residual vector $y - \hat{y}_0$ for all of $T_{OmniK}^M$, $T_{OmniK}^I$ and $T_{OmniK}^{B}$, simultaneously, as in Eq. (19).


\begin{eqnarray*} P_{OmniK(r^{\prime })}^{M} = \frac{1}{R - 1} \sum _{r^{\prime } \ne r} I(T_{OmniK}^{M(r)} \le T_{OmniK}^{M(r^{\prime })}), \end{eqnarray*}

\begin{eqnarray*} P_{OmniK(r^{\prime })}^{I} = \frac{1}{R - 1} \sum _{r^{\prime } \ne r} I(T_{OmniK}^{I(r)} \le T_{OmniK}^{I(r^{\prime })}), \end{eqnarray*}

\begin{eqnarray*} P_{OmniK(r^{\prime })}^{B} = \frac{1}{R - 1} \sum _{r^{\prime } \ne r} I(T_{OmniK}^{B(r)} \le T_{OmniK}^{B(r^{\prime })}), \end{eqnarray*}

and


\begin{eqnarray*} T_{OmniK(r^{\prime })} = \min \lbrace P_{OmniK(r^{\prime })}^{M}, P_{OmniK(r^{\prime })}^{I}, P_{OmniK(r^{\prime })}^{B}\rbrace , \end{eqnarray*}

which is equivalently,


\begin{eqnarray*} T_{OmniK(r^{\prime })} &=& \min [\min _{h \in \Gamma } \lbrace \frac{1}{R - 1} \sum _{r^{\prime } \ne r} I(T_{(h)}^{M(r)} \ge T_{(h)}^{M(r^{\prime })})\rbrace , \nonumber \\ && \min _{h \in \Gamma } \lbrace \frac{1}{R - 1} \sum _{r^{\prime } \ne r} I(T_{(h)}^{I(r)} \ge T_{(h)}^{I(r^{\prime })})\rbrace , \end{eqnarray*}

(19) \begin{eqnarray*} \min _{h \in \Gamma } \lbrace \frac{1}{R - 1} \sum _{r^{\prime } \ne r} I(T_{(h)}^{B(r)} \ge T_{(h)}^{B(r^{\prime })})\rbrace ]. \end{eqnarray*}

where *R* is the number of rearrangements with *r* = 1, …, *R* and *r*′ = 1, …, *R*. Then, we can calculate a *P*-value for OmniK as the proportion of null test statistic values smaller than or equal to the observed test statistic value as in Eq. (20).


(20) \begin{eqnarray*} P_{OmniK} = \frac{1}{R} \sum _{r^{\prime } = 1}^{R} I(T_{OmniK(r^{\prime })} \le T_{OmniK(obs)}), \end{eqnarray*}

Note that *T*_*OmniK*_ reflects the strongest evidence of significance across all the endogenous and input kernels. Therefore, OmniK can robustly maintain a high test power whenever the main effects, interaction effects, or both of them exist and also for a variety of linear or nonlinear patters of the relationships suited by multiple input kernels. OmniK is also computationally efficient because the score test statistic itself does not require any iterative calculations and also the null test statistic values, $T_{OmniK}^{M(r)}$’s, $T_{OmniK}^{I(r)}$’s, $T_{OmniK}^{B(r)}$’s, $T_{OmniK}^{M(r^{\prime })}$’s, $T_{OmniK}^{I(r^{\prime })}$’s and $T_{OmniK}^{B(r^{\prime })}$’s, can be repeatedly employed with no double iterative calculations. Therefore, it is promising to accommodate more input kernels for more intensive learning processes to be conducted.

## Results

### Simulation settings

I conducted simulation experiments to compare my proposed methods with other existing methods, CKLRT (17) and CKAT (18), using a parametric bootstrap method based on the Dirichlet-multinomial distribution (29) as in (7). To reflect real microbiome composition, I estimated its parameters on the proportions and over-dispersion (29) using the Charlson et al’s upper-respiratory-tract microbiome data (30). Then, I used the Dirichlet-multinomial distribution (29) specified with the estimated parameters and the total count of 10 000 to generate microbial counts. I surveyed two sample sizes of 100 (*n* = 100) and 200 (*n* = 200), respectively. Then, I generated continuous responses based on Eq. (21) and binary responses based on Eq. (22).


(21) \begin{eqnarray*} y = \alpha _1 + T \alpha _2 + Z \alpha _3 + f(X_S) \beta _1 + (f(X_S) \odot T) \beta _2 + \epsilon , \, \end{eqnarray*}

and


(22) \begin{eqnarray*} \text{logit}(E(y|X, T, Z)) &=& \alpha _1 + T \alpha _2 + Z \alpha _3 + f(X_S) \beta _1 \nonumber \\ && + \, (f(X_S) \odot T) \beta _2, \end{eqnarray*}

where *f*(·) is a basis function for a linear or non-linear transformation; α_1_ is a scalar for the intercept (α_1_ = 0); *T* is an *n* × 1 vector for a treatment, and α_2_ is a scalar for its main effect (α_2_ = 1); *Z* is an *n* × 2 matrix for two covariates, *z*_1_ and *z*_2_, that are independent to variants: *z*_1_ ∼ *Bern*(0.5) and correlated with 10% of randomly selected variants (*X*′): *z*_2_ ∼ *X*′ · 0.5 + *N*(0, 1), respectively, and α_3_ is a 2 × 1 vector for their main effects (α_3_ = 0.5_2 × 1_); *f*(*X*_*S*_) is an *n* × *p*′ matrix for a subset of variants with *p*′ < *p*, and β_1_ is a *p*′ × 1 vector for their main effects; *f*(*X*_*S*_)⊙*T* is an *n* × *p*′ matrix for the Hadamard products (i.e. interaction terms) between *f*(*X*_*S*_) and *T*, and β_2_ is a *p*′ × 1 vector for their interaction effects; and ε is a random error: ε ∼ *N*(0, 1). To mimic a randomized clinical trial, where a treatment assignment is made randomly, I generated *T* to be independent: *T* ∼ *Bern*(0.5), while to mimic an observational study, I generated *T* to be dependent to *Z*: *T* ∼ *Bern*(1/(1 + *exp*( − *f*(*Z*) · 0.5_2 × 1_)).

To estimate type I error rates, I set both of the main and interaction effects to be absent: $\beta _1 = 0_{p^{\prime } \times 1}$ and $\beta _2 = 0_{p^{\prime } \times 1}$. To estimate power values, I first selected a subset of variants, *X*_*S*_, to be phylogenetically related. Specifically, they are the variants in a cluster among five clusters partitioned by the partitioning-around-medoids (PAM) algorithm (31) based on variant-by-variant cophenetic distances (32). Then, I set (i) the main effects to be present as $\beta _1 = 2_{p^{\prime } \times 1}$, but the interaction effects to be absent as $\beta _2 = 0_{p^{\prime } \times 1}$; (ii) the main effects to be absent as $\beta _1 = 0_{p^{\prime } \times 1}$, but the interaction effects to be present as $\beta _2 = 1_{p^{\prime } \times 1}$ for Eq. (21) and $\beta _2 = 3_{p^{\prime } \times 1}$ for Eq. (22); and (iii) both of the main and interaction effects to be present as $\beta _1 = 2_{p_1^{\prime } \times 1}$ and $\beta _2 = -1_{p_2^{\prime } \times 1}$ for Eq. (21) and $\beta _1 = -1.5_{p_1^{\prime } \times 1}$ and $\beta _2 = 3_{p_2^{\prime } \times 1}$ for Eq. (22) with $p^{\prime } = p_1^{\prime } + p_2^{\prime }$. In the meantime, to survey a linear relationship, I set *f*(·) to be the standardization function for the variants to have mean 0 and variance 1, while to survey a non-linear discrete relationship, I set *f*(·) to be the indicator function for the variants to have 0 for absence and 1 for presence.

For OmniK, I employed the Jaccard, Bray-Curtis, unweighted UniFrac, generalized UniFrac (0.25), generalized UniFrac (0.5), generalized UniFrac (0.75) and weighted UniFrac kernels, denoted as, *K*_*J*_, *K*_*BC*_, *K*_*U*_, *K*_0.25_, *K*_0.5_, *K*_0.75_ and *K*_*W*_, as candidate kernels in Γ with the number of randomly selected rearrangements to be 3000 (*R* = 3000). For CKLRT (17), I used the R package, CKLRT.0.2.3 (https://cran.r-project.org/web/packages/CKLRT/) with the default settings based on the likelihood ratio test (LRT) and restricted likelihood ratio test (RLRT), respectively. For CKAT (18), I used the R package, CKAT.0.1.0 (https://cran.r-project.org/web/packages/CKAT/), with the default settings based on the linear kernel (say, Linear) and quadratic kernel (say, Quadratic), respectively.

For additional references, I surveyed the degrees-of-freedom of 10 (*df* = 10), 20 (*df* = 20) and 30 (*df* = 30) as well as the full degrees of freedom that I mainly propose. I also surveyed other omnibus testing methods of the Fisher’s method (33), Brown’s method (34) and Simes’ method (35) as well as the minimum *P*-value approach (19) that I propose for OmniK. Finally, I used Yanai et al’s gut microbiome data (26) (see *Applications to real microbiome data: gut microbiome and its interaction with a diet method on body weights* for more details) to estimate the parameters on the proportions and over-dispersion of the Dirichlet-multinomial distribution (29), and then conducted the above simulation experiments all over again. This is to survey possible disparities in simulation results between the upper-respiratory-tract microbiome data (30) and the gut microbiome data (26).

### Simulation results

#### Type 1 error.

I found empirical type I error rates under the significance level of 5% for all the surveyed methods, except for CKLRT (17) based on LRT and RLRT, for both sample sizes of *n* = 100 and *n* = 200, both continuous and binary responses, and both randomized clinical trial and observational study (Table 1). Hence, I excluded CKLRT (17) based on LRT and RLRT only for later test power comparisons and real data applications. I also noted lower empirical type I error rates for binary responses than continuous responses (Table 1). Especially, the omnibus testing approach for interaction effects (i.e. OmniK (I)) makes it more conservative than the ones for main effects (i.e. OmniK (M)) and both of them (i.e., OmniK (B)) (Table 1). However, such conservativeness is not an issue that is unique to my proposed methods. That is, CKAT (18) also reports low empirical type I error rates for the binary responses (Table 1). The reason can be because of the discrete nature of binary responses, which makes it harder to reflect the interaction effects to binary responses than continuous responses.

**Table 1.** Empirical type 1 error rates (unit: %) estimated using (i) existing methods: CKLRT (17) based on LRT and RLRT, respectively (see LRT and RLRT), and CKAT (18) based on linear and quadratic kernels, respectively (see Linear and Quadratic); (ii) general kernel machine regression analysis for each ecological kernel (see *K*_*J*_, *K*_*BC*_, *K*_*U*_, *K*_0.25_, *K*_0.5_, *K*_0.75_ and *K*_*W*_); (iii) omnibus testing approach for each endogenous kernel on main effects, interaction effects or both of them (see OmniK (M), OmniK (I), OmniK (B)) and (iv) omnibus testing approach across all endogenous and input kernels (see OmniK). * The parameters of the Dirichlet-multinomial distribution were estimated using the Charlson et al’s upper-respiratory-tract microbiome data (30). * CR (R) represents continuous response and randomized clinical trial; CR (O) represents continuous response and observational study; BR (R) represents binary response and randomized clinical trial; BR (O) represents binary response and observational study. * CKLRT (17) is available for continuous response only

*n* = 100				
Method	CR (R)	CR (O)	BR (R)	BR (O)
LRT	9.44	10.27	-	-
RLRT	7.04	7.70	-	-
Linear	1.97	1.94	1.45	0.02
Quadratic	4.27	4.11	3.08	0.56
*K*_*J*_	4.93	5.10	3.68	3.63
*K*_*BC*_	4.78	5.05	3.89	3.85
*K*_*U*_	5.02	5.05	3.85	3.88
*K*_0.25_	4.95	5.10	3.67	3.71
*K*_0.5_	4.95	4.99	3.84	3.71
*K*_0.75_	4.96	5.05	4.05	3.88
*K*_*W*_	4.96	4.91	4.19	3.93
OmniK (M)	4.76	4.88	4.92	4.90
OmniK (I)	5.00	5.03	2.73	2.66
OmniK (B)	4.96	5.01	3.43	3.23
OmniK	4.98	5.07	3.84	3.87
*n* = 200				
LRT	7.86	6.89	-	-
RLRT	6.33	5.58	-	-
Linear	4.01	3.99	0.16	0.16
Quadratic	5.08	4.89	0.55	0.91
*K*_*J*_	5.06	4.91	3.76	3.64
*K*_*BC*_	4.98	4.94	3.83	3.77
*K*_*U*_	5.09	4.91	3.69	3.74
*K*_0.25_	4.92	4.81	3.74	3.68
*K*_0.5_	4.90	4.68	3.72	3.81
*K*_0.75_	4.94	4.80	3.88	3.86
*K*_*W*_	4.96	4.77	4.05	3.88
OmniK (M)	5.02	4.76	4.79	4.76
OmniK (I)	4.89	4.92	2.58	2.47
OmniK (B)	4.95	4.86	3.20	3.03
OmniK	5.10	4.84	3.81	3.81

For additional references, I found similar empirical type I error rates across all the surveyed degrees-of-freedom: OmniK with 10 *df*, OmniK with 20 *df*, OmniK with 30 *df*, and OmniK with full *df* (Table 2). I also found highly inflated empirical type I error rates for other omnibus testing approaches of the Fisher’s method (33), Brown’s method (34) and Simes’ method (35) (Table 2). Finally, I organized empirical type I error rates based on the gut microbiome data (26) in *Supplementary Data*: S1 Table and S2 Table, and found similar results with the same conclusions in test validity and conservativeness.

**Table 2.** Empirical type 1 error rates (unit: %) estimated using (i) OmniK with 10 *df*, OmniK with 20 *df*, OmniK with 30 *df* and OmniK with full *df*; and (ii) other omnibus testing methods of the Fisher’s method (33), Brown’s method (34) and Simes’ method (35) * The parameters of the Dirichlet-multinomial distribution were estimated using the Charlson et al’s upper-respiratory-tract microbiome data (30). * CR (R) represents continuous response and randomized clinical trial; CR (O) represents continuous response and observational study; BR (R) represents binary response and randomized clinical trial; BR (O) represents binary response and observational study

*n* = 100				
Method	CR (R)	CR (O)	BR (R)	BR (O)
OmniK: 10	5.01	5.17	3.77	3.81
OmniK: 20	4.98	4.97	3.83	3.82
OmniK: 30	5.09	4.99	3.90	3.90
OmniK: full	4.98	5.07	3.84	3.87
Fisher	22.72	22.96	15.52	15.42
Brown	8.22	8.39	6.23	6.23
Simes	20.01	20.07	15.54	15.41
*n* = 200				
OmniK: 10	4.97	5.00	3.74	3.83
OmniK: 20	5.09	5.02	3.94	3.89
OmniK: 30	4.99	5.03	3.90	3.90
OmniK: full	5.10	4.84	3.81	3.81
Fisher	22.10	22.09	15.59	15.98
Brown	8.32	8.90	6.30	6.29
Simes	20.09	19.92	15.21	15.53

#### Power.

**Figure 1.**
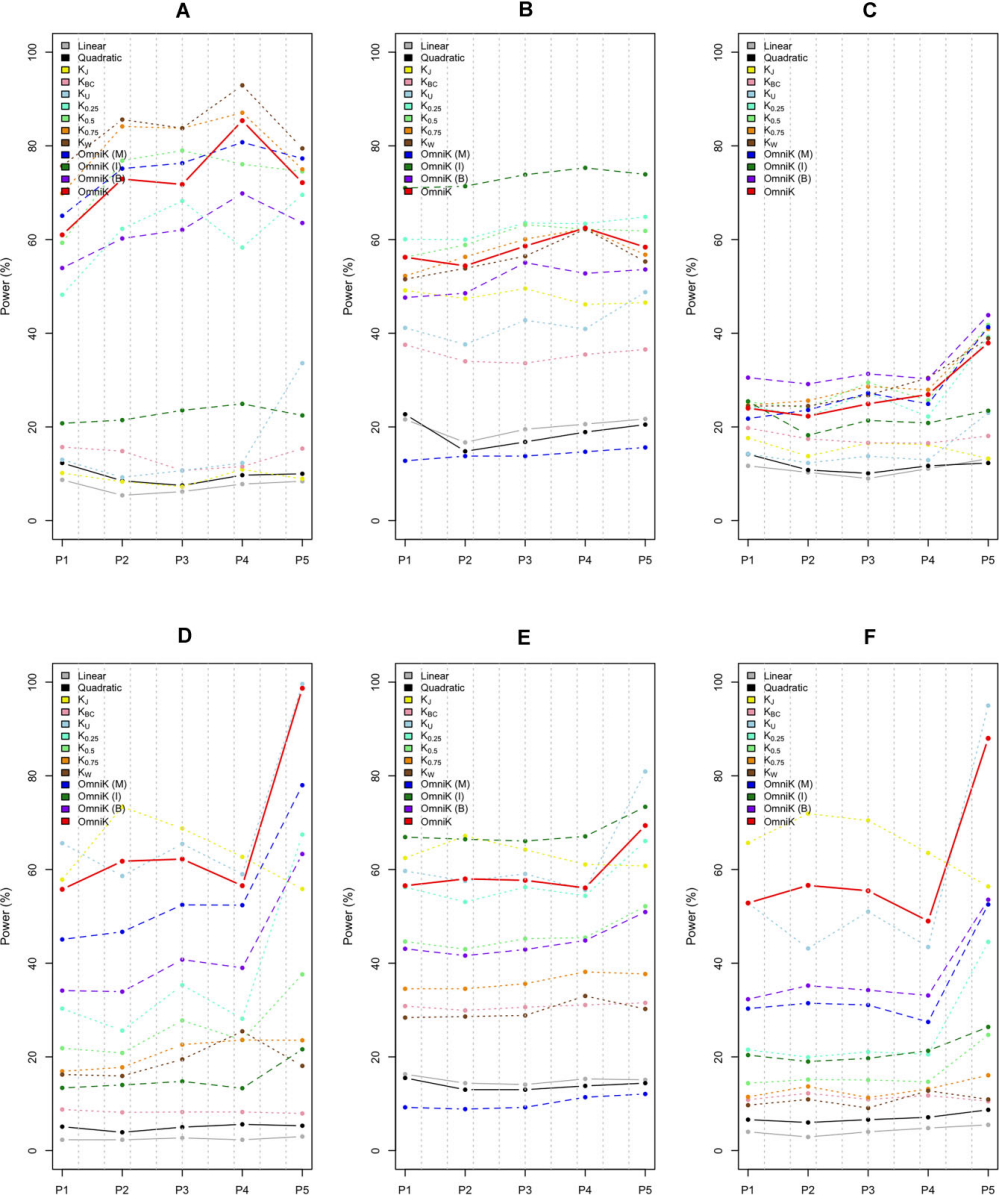
Empirical powers for continuous response and randomized clinical trial (*n* = 100) using (i) existing methods: CKAT based on linear and quadratic kernels, respectively (see Linear and Quadratic); (ii) general kernel machine regression analysis for each ecological kernel (see *K*_*J*_, *K*_*BC*_, *K*_*U*_, *K*_0.25_, *K*_0.5_, *K*_0.75_ and *K*_*W*_); (iii) omnibus testing approach for each endogenous kernel on main effects, interaction effects or both of them (see OmniK (M), OmniK (I), OmniK (B)) and (iv) omnibus testing approach across all endogenous and input kernels (see OmniK). * The parameters of the Dirichlet-multinomial distribution were estimated using the Charlson et al’s upper-respiratory-tract microbiome data (30). * (**A**) is for linear relationship with main effects; (**B**) is for linear relationship with interaction effects; (**C**) is for linear relationship with both of main and interaction effects; (**D**) is for nonlinear discrete relationship with main effects; (**E**) is for nonlinear discrete relationship with interaction effects; (**F**) is for nonlinear discrete relationship with both of main and interaction effects. * P1–P5 represents a selected phylogenetic cluster.

**Figure 2.**
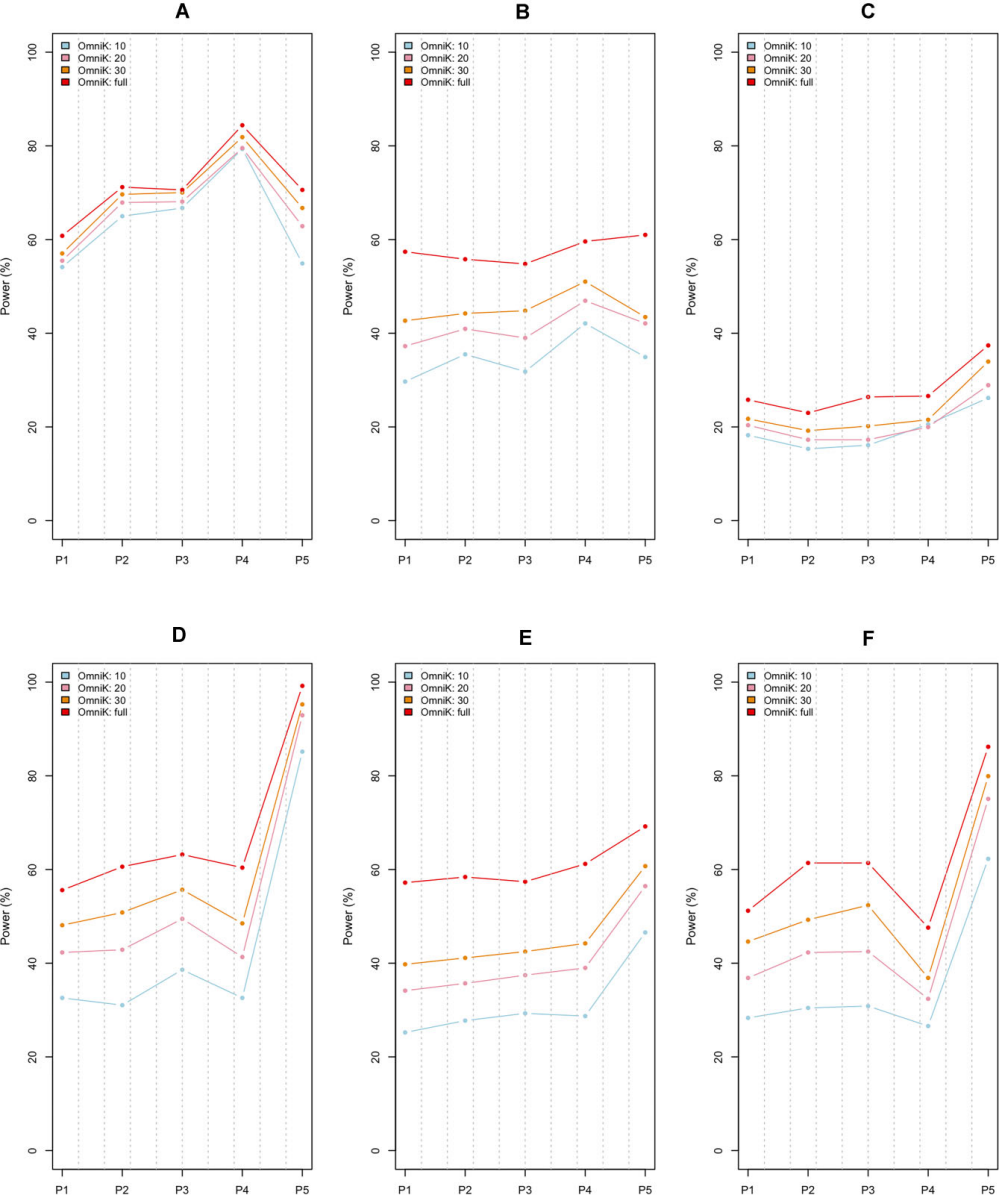
Empirical powers for continuous response and randomized clinical trial (*n* = 100) using OmniK with 10 *df*, OmniK with 20 *df*, OmniK with 30 *df* and OmniK with full *df*. * The parameters of the Dirichlet-multinomial distribution were estimated using the Charlson et al’s upper-respiratory-tract microbiome data (30). * (**A**) is for linear relationship with main effects; (**B**) is for linear relationship with interaction effects; (**C**) is for linear relationship with both of main and interaction effects; (**D**) is for nonlinear discrete relationship with main effects; (**E**) is for nonlinear discrete relationship with interaction effects; (**F**) is for nonlinear discrete relationship with both of main and interaction effects. * P1–P5 represents a selected phylogenetic cluster.

I reported empirical power values for the continuous response and the randomized clinical trial with the sample size of *n* = 100 in Figure 1 using (i) the existing methods: CKAT based on the linear and quadratic kernels, respectively (see linear and quadratic); (ii) the general kernel machine regression analysis for each ecological kernel (see *K*_*J*_, *K*_*BC*_, *K*_*U*_, *K*_0.25_, *K*_0.5_, *K*_0.75_ and *K*_*W*_); (iii) the omnibus testing approach for each endogenous kernel on the main effects, interaction effects or both of them (see OmniK (M), OmniK (I), OmniK (B)) and (iv) the omnibus testing approach across all the endogenous and input kernels (see OmniK). I also reported the empirical power values for the continuous response and the randomized clinical trial with the sample size of *n* = 100 in Figure 2 using (i) OmniK with 10 *df*, OmniK with 20 *df*, OmniK with 30 *df* and OmniK with full *df*. To save space, I moved all the other results to *Supplementary Data*: (i) S1 Figure and S2 Figure are for the continuous response and the randomized clinical trial with the sample size of *n* = 200; (ii) S3 Figure and S4 Figure are for the continuous response and the observational study with the sample size of *n* = 100; (iii) S5 Figure and S6 Figure are for the continuous response and the observational study with the sample size of *n* = 200; (iv) S7 Figure and S8 Figure are for the binary response and the randomized clinical trial with the sample size of *n* = 100; (v) S9 Figure and S10 Figure are for the binary response and the randomized clinical trial with the sample size of *n* = 200; (vi) S11 Figure and S12 Figure are for the binary response and the observational study with the sample size of *n* = 100 and (vii) S13 Figure and S14 Figure are for the binary response and the observational study with the sample size of *n* = 200. Finally, I organized empirical power values based on the gut microbiome data (26) in *Supplementary Data*: from S15 Figure to S30 Figure.

First, for the linear relationships (Figure 1A–C and S1, S3, S5, S7, S9, S11, S13 Figure: A–C), I found higher empirical power values for the abundance-based kernels (e.g., *K*_*W*_, *K*_0.25_, *K*_0.5_, *K*_0.75_, *K*_*BC*_) than the presence-absence-based kernels (e.g., *K*_*U*_, *K*_*J*_), as expected. For the nonlinear discrete relationships (Figure 1D–F and S1, S3, S5, S7, S9, S11, S13 Figure: D–F), I found higher empirical power values for the presence-absence-based kernels (e.g. *K*_*U*_, *K*_*J*_) than the abundance-based kernels (e.g. *K*_*W*_, *K*_0.25_, *K*_0.5_, *K*_0.75_, *K*_*BC*_), as expected. Above all, I found robustly high empirical power values for OmniK for either the linear relationships (Figure 1A–C and S1, S3, S5, S7, S9, S11, S13 Figure: A–C) or the nonlinear discrete relationships (Figure 1D–F and S1, S3, S5, S7, S9, S11, S13 Figure: D–F), which because of its adaptivity across the input kernels.

Second, for only the main effects to be present (Figure 1A, D and S1, S3, S5, S7, S9, S11, S13 Figure: A, D), I found higher empirical power values for OmniK (M) based on the main effect endogenous kernel than OmniK (I) based on the interaction effect endogenous kernel and OmniK (B) based on the both main and interaction effect endogenous kernel, as expected. For only the interaction effects to be present (Figure 1B, E and S1, S3, S5, S7, S9, S11, S13 Figure: B, E), I found higher empirical power values for OmniK (I) than OmniK (M) and OmniK (B), as expected. For both of the main and interaction effects to be present (Figure 1C, F and S1, S3, S5, S7, S9, S11, S13 Figure: C, F), I found higher empirical power values for OmniK (B) than OmniK (M) and OmniK (I), as expected. Above all, I found robustly high empirical power values for OmniK for either of only the main effects to be present (Figure 1A, D and S1, S3, S5, S7, S9, S11, S13 Figure: A, D), only the interaction effects to be present (Figure 1B, E and S1, S3, S5, S7, S9, S11, S13 Figure: B, E), or both of the main and interaction effects to be present (Figure 1C, F and S1, S3, S5, S7, S9, S11, S13 Figure: C, F). I also found that OmniK is more powerful than CKAT (18) based on the linear or quadratic kernel for all surveyed scenarios (Figure 1 and S1, S3, S5, S7, S9, S11, S13 Figure).

For additional references, I found lower empirical power values for lower degrees-of-freedom: OmniK with 10 *df* < OmniK with 20 *df* < OmniK with 30 *df* < OmniK with full *df* (Figure 2 and S2, S4, S6, S8, S10, S12, S14 Figure). Finally, I found similar results with the same conclusions in relative power performance for the use of gut microbiome data (from S15 Figure to S30 Figure). I also noted different empirical power values across different phylogenetic clusters: P1-P5 (Figures 1–2, S1–S30 Figure). There are indeed numerous factors that can influence the power performance, such as phylogenetic closeness, rareness, skewness, nonlinearity and so forth, with different degrees and combinations. Hence, it is extremely hard to predict the power performance for each cluster precisely. Nonetheless, OmniK maintains a high power performance with high adaptivity, which is the key point of this research.

### Applications to real microbiome data

#### Gut microbiome and its interaction with a diet method on body weights

I utilized my proposed and other existing methods to survey the roles of the gut microbiome and its interaction with a diet method on body weight. For this, I employed the public gut microbiome data published in Yanai *et al.* (26). Yanai *et al.* (26) recruited 23 rhesus monkeys, between 7 and 14 years in age, maintained and housed at the National Institute of Health Animal Center. Yanai *et al.* randomly assigned the rhesus monkeys to two different diet methods, ad-libitum and periodically restricted feeding (PRF); more specifically, 11 rhesus monkeys to ad-libitum and 12 rhesus monkeys to PRF. Their gut microbiomes were profiled at the baseline using 16S rRNA sequencing, and their body weights were measured two weeks after the diet (26). I added age and sex as covariates in the analysis. I set the number of randomly selected rearrangements to be 300 000 (*R* = 300 000).

I found the smallest *P*-value of 0.093 for *K*_*BC*_ with respect to input kernels (Table 3: Study 1), which may indicate that non-phylogenetic common variants, rather than rare variants, in the gut microbiome influence body weights. I also found the smallest *P*-value of 0.127 for OmniK (B) with respect to endogenous kernels (Table 3: Study 1), which may indicate that the gut microbiome influences body weights both directly and indirectly through main and interaction effects. Overall, across all the input and endogenous kernels, the *P*-value for OmniK is 0.206 (Table 3: Study 1). CKAT based on the linear and quadratic kernels returned the *P*-values of 0.482 and 0.598 (Table 3: Study 1). None of my proposed and other existing methods returned statistically significance at the level of 0.05 (Table 3: Study 1), which might be because of the small sample size of 23.

**Table 3.** The *P*-values estimated using (i) existing methods: CKAT (18) based on the linear and quadratic kernels, respectively (see Linear and Quadratic); (ii) general kernel machine regression analysis for each ecological kernel (see *K*_*J*_, *K*_*BC*_, *K*_*U*_, *K*_0.25_, *K*_0.5_, *K*_0.75_ and *K*_*W*_); (iii) omnibus testing approach for each endogenous kernel on main effects, interaction effects or both of them (see OmniK (M), OmniK (I), OmniK (B)); and (iv) the omnibus testing approach across all endogenous and input kernels (see OmniK). * Study 1 is the gut microbiome and its interaction with a diet method on body weights; Study 2 is for oral microbiome and its interaction with e-cigarette smoking on gingival inflammation

Method	Study 1	Study 2
Linear	0.482	0.835
Quadratic	0.598	0.813
*K*_*J*_	0.465	0.007
*K*_*BC*_	0.093	0.563
*K*_*U*_	0.581	0.017
*K*_0.25_	0.188	0.412
*K*_0.5_	0.162	0.773
*K*_0.75_	0.159	0.890
*K*_*W*_	0.143	0.915
OmniK (M)	0.145	0.021
OmniK (I)	0.207	0.030
OmniK (B)	0.127	0.013
OmniK	0.206	0.024

#### Oral microbiome and its interaction with E-cigarette use on gingival inflammation

I also utilized my proposed and other existing methods to survey the roles of the oral microbiome and its interaction with e-cigarette use on gingival inflammation. For this, I employed the public oral microbiome data published in Park *et al.* (27). Park *et al.* (27) recruited 145 participants, between 18 and 34 years in age, from Baltimore, Maryland and its surrounding areas. Park *et al.* (27) observed the participants as 74 non-users and 71 e-cigarette users. Their oral microbiomes in salivary niches were profiled using 16S rRNA sequencing, and their gingival inflammation status was measured as 0 for no inflammation and 1 for the presence of inflammation (27). I added age and sex as covariates in the analysis. I set the number of randomly selected rearrangements to be 300 000 (*R* = 300 000).

I found the significant *P*-values of 0.007 and 0.017 for *K*_*J*_ and *K*_*U*_, respectively, at the level of 0.05 with respect to input kernels (Table 3: Study 2), which may indicate that non-phylogenetic or phylogenetic rare variants, rather than common variants, in the oral microbiome influence gingival inflammation. I also found the significant *P*-values of 0.021, 0.030 and 0.013 for OmniK (M), OmniK (I) and OmniK (B), respectively, with respect to endogenous kernels (Table 3: Study 2), which may indicate that the oral microbiome influences gingival inflammation directly and/or indirectly through main and/or interaction effects. Overall, across all the input and endogenous kernels, the *P*-value for OmniK is 0.024 (Table 3: Study 2). Though, CKAT based on the linear and quadratic kernels returned non-significant *P*-values of 0.835 and 0.813 (Table 3: Study 2).

## Discussion

In this paper, I introduced a general kernel machine regression framework using principal component analysis for jointly testing main and interaction effects. It begins with extracting principal components from an input kernel through the singular value decomposition. Then, it employs the principal components as surrogate variants for the underlying real variants to construct three endogenous kernels for the (i) main effects, (ii) interaction effects and (iii) both of the main and interaction effects, respectively. Hence, it works with a kernel as an input without knowing its underlying real variants, while the other existing methods, CKLRT (17) and CKAT (18), do not. It also detects either the main effects, interaction effects, or both of them robustly through omnibus testing across the three endogenous kernels. I also introduced its omnibus testing extension to multiple input kernels, named as OmniK, for a unified and powerful inference across multiple input kernels, while CKLRT (17) and CKAT (18) can process multiple input kernels only individually. I also revealed its outperformance in significance testing, compared with CKLRT (17) and CKAT (18), through extensive simulation experiments. I also applied it to two real microbiome datasets on (i) the gut microbiome and its interaction with a diet method on body weight for rhesus monkeys (26); and (ii) the oral microbiome and its interaction with e-cigarette smoking on gingival inflammation (27).

I demonstrated OmniK using ecological kernels, such as Jaccard (21), Bray-Curtis (22), unweighted UniFrac (23), generalized UniFrac (24) and weighted UniFrac (25) kernels, in human microbiome studies. However, I do not restrict its use to ecological kernels. OmniK is a general framework that can accept any kernels as inputs; hence, its methodology can apply to various disciplines. Of course, its performance can depend on which kernels are used. The ecological kernels that I used are suited to human microbiome studies; yet, there can be better kernels for other disciplines. There is also a strong need for developing new kernels for better performances.

Finally, the interaction effects on which I have focused are the ones between genetic or microbial variants and a treatment (e.g. medical treatment, environmental factor, health policy). The use of phylogenetic kernels (e.g. unweighted UniFrac (23), generalized UniFrac (24) and weighted UniFrac (25)) and the simultaneous rearrangements of the residual vector for different kernels can account for possible phylogenetic and compositional correlations across variants; yet, they are not directly about the variant-by-variant interactions. It is also crucial to address the variant-by-variant interactions, and the use of surrogate variants can also promise explicit regression modelling with the second- and higher-order terms. However, it is also challenging to deal with numerous possible second- and higher-order interaction terms simultaneously. Further research is needed. I could not satisfy all demands in this study.

## Supplementary Material

lqae148_Supplemental_File

## Data Availability

I used two public real microbiome datasets on (i) the gut microbiome and its interaction with a diet method on body weight for rhesus monkeys (26), for which the raw sequence data are deposited in the NCBI Gene Expression Omnibus database (http://www.ncbi.nlm.nih.gov/geo/) under accession number GSE235769 and (ii) the oral microbiome and its interaction with e-cigarette smoking on gingival inflammation (27), for which the raw sequence data are deposited in the NCBI Gene Expression Omnibus database (http://www.ncbi.nlm.nih.gov/geo/) under accession number GSE201949. Their processed datasets are also available in the R package, OmniK (10.6084/m9.figshare.27252075), with three R objects: gut.otu.table, gut.tree and gut.meta for the first dataset and oral.otu.table, oral.tree and oral.meta for the second dataset. OmniK is freely available in the R package, OmniK (10.6084/m9.figshare.27252075), including detailed documentation to assist users on installation, inputs, options, and outputs with example data and programs.
