# Supplementary material for: A general kernel machine regression framework using principal component analysis for jointly testing main and interaction effects: Applications to human microbiome studies
Source: NAR Genom Bioinform. 2024 Nov 12;6(4):lqae148. doi: 10.1093/nargab/lqae148 (PMC11555437; doi:10.1093/nargab/lqae148)
Supplement: lqae148_Supplemental_File [file lqae148_supplemental_file.pdf]

SUPPLEMENTARY DATA: A GENERAL KERNEL MACHINE REGRESSION FRAMEWORK USING PRINCIPAL COMPONENT ANALYSIS FOR JOINTLY TESTING MAIN AND INTERACTION EFFECTS: APPLICATIONS TO HUMAN MICROBIOME STUDIES

Hyunwook Koh

Department of Applied Mathematics and Statistics
The State University of New York, Korea
Incheon, South Korea
hyunwook.koh@stonybrook.edu

S1 Table: Empirical type 1 error rates (unit: %) estimated using (i) existing methods: CKLRT based on LRT and RLRT, respectively (see LRT and RLRT), and CKAT based on linear and quadratic kernels, respectively (see Linear and Quadratic); (ii) general kernel machine regression analysis for each ecological kernel (see K_J, K_{BC}, K_U, $K_{0.25}$, $K_{0.5}$, $K_{0.75}$ and K_W); (iii) omnibus testing approach for each endogenous kernel on main effects, interaction effects or both of them (see OmniK (M), OmniK (I), OmniK (B)); and (iv) omnibus testing approach across all endogenous and input kernels (see OmniK). * The parameters of the Dirichlet-multinomial distribution were estimated using the Yanai et al's gut microbiome data. * CR (R) represents continuous response and randomized clinical trial; CR (O) represents continuous response and observational study; BR (R) represents binary response and randomized clinical trial; BR (O) represents binary response and observational study. * CKLRT is available for continuous response only.

$n = 100$					$n = 200$				
Method	CR (R)	CR (O)	BR (R)	BR (O)	Method	CR (R)	CR (O)	BR (R)	BR (O)
LRT	9.88	10.12	-	-	LRT	9.03	10.27	-	-
RLRT	7.14	7.80	-	-	RLRT	7.04	7.62	-	-
Linear	1.87	2.04	1.51	0.03	Linear	1.97	1.94	1.45	0.02
Quadratic	4.37	4.18	3.12	0.57	Quadratic	4.27	4.11	3.08	0.56
K_J	4.95	5.01	3.72	3.70	K_J	4.93	5.10	3.68	3.63
K_{BC}	4.79	5.01	3.82	3.80	K_{BC}	4.78	5.05	3.89	3.85
K_U	5.12	5.02	3.84	3.98	K_U	5.02	5.05	3.85	3.88
$K_{0.25}$	4.99	5.00	3.77	3.81	$K_{0.25}$	4.95	5.10	3.67	3.71
$K_{0.5}$	5.01	4.89	3.94	3.79	$K_{0.5}$	4.95	4.99	3.84	3.71
$K_{0.75}$	4.92	4.95	4.15	3.84	$K_{0.75}$	4.96	5.05	4.05	3.88
K_W	5.00	5.11	4.08	3.94	K_W	4.96	4.91	4.19	3.93
OmniK (M)	4.86	4.89	4.99	4.92	OmniK (M)	4.76	4.88	4.92	4.90
OmniK (I)	5.09	5.00	2.76	2.76	OmniK (I)	5.00	5.03	2.73	2.66
OmniK (B)	4.99	5.00	3.51	3.63	OmniK (B)	4.96	5.01	3.43	3.23
OmniK	5.00	5.02	3.90	3.83	OmniK	4.98	5.07	3.84	3.87

S2 Table: Empirical type 1 error rates (unit: %) estimated using (i) OmniK with 10 df, OmniK with 20 df, OmniK with 30 df and OmniK with full df; and (ii) other omnibus testing methods of the Fisher's method, Brown's method and Simes' method * The parameters of the Dirichlet-multinomial distribution were estimated using the Yanai et al's gut microbiome data. * CR (R) represents continuous response and randomized clinical trial; CR (O) represents continuous response and observational study; BR (R) represents binary response and randomized clinical trial; BR (O) represents binary response and observational study.

$n = 100$					$n = 200$				
Method	CR (R)	CR (O)	BR (R)	BR (O)	Method	CR (R)	CR (O)	BR (R)	BR (O)
OmniK: 10	5.03	5.09	3.87	3.90	OmniK: 10	4.97	5.00	3.74	3.83
OmniK: 20	5.06	4.88	3.77	3.83	OmniK: 20	5.09	5.02	3.94	3.89
OmniK: 30	5.02	4.98	3.89	3.99	OmniK: 30	4.99	5.03	3.90	3.90
OmniK: full	5.00	5.02	3.90	3.83	OmniK: full	4.98	5.07	3.84	3.87
Fisher	22.72	22.96	15.52	15.42	Fisher	23.33	24.00	15.71	15.57
Brown	8.22	8.39	6.23	6.23	Brown	8.21	8.55	6.31	6.30
Simes	20.01	20.07	15.54	15.41	Simes	20.10	20.09	15.09	15.08

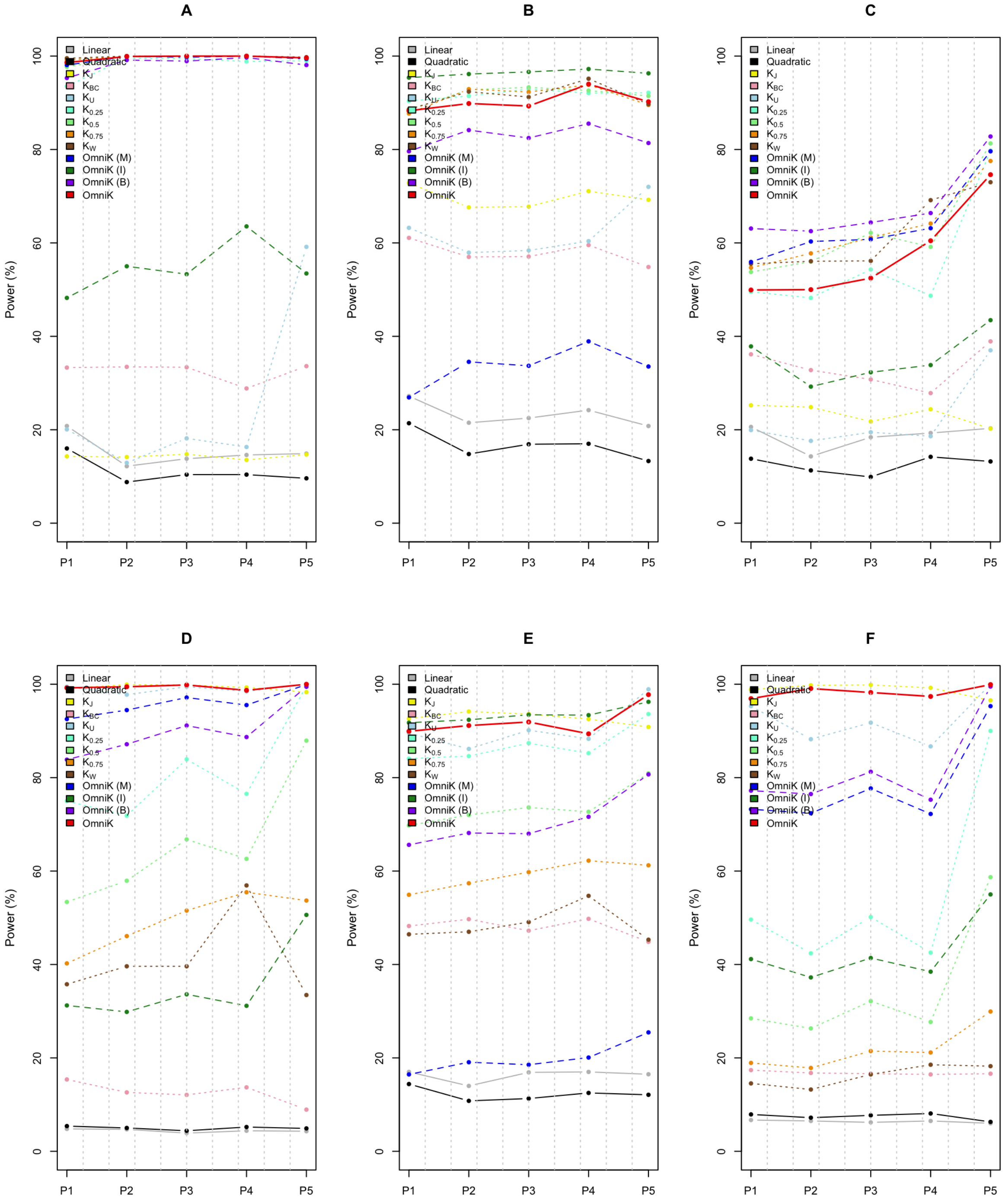

S1 Figure: Empirical powers for continuous response and randomized clinical trial ($n = 200$) using (i) existing methods: CKAT based on linear and quadratic kernels, respectively (see Linear and Quadratic); (ii) general kernel machine regression analysis for each ecological kernel (see K_J, K_{BC}, K_U, $K_{0.25}$, $K_{0.5}$, $K_{0.75}$ and K_W); (iii) omnibus testing approach for each endogenous kernel on main effects, interaction effects or both of them (see OmniK (M), OmniK (I), OmniK (B)); and (iv) omnibus testing approach across all endogenous and input kernels (see OmniK). * The parameters of the Dirichlet-multinomial distribution were estimated using the Charlson et al's upper-respiratory-tract microbiome data.* A is for linear relationship with main effects; B is for linear relationship with interaction effects; C is for linear relationship with both of main and interaction effects; D is for nonlinear discrete relationship with main effects; E is for nonlinear discrete relationship with interaction effects; F is for nonlinear discrete relationship with both of main and interaction effects. * P1-P5 represents a selected phylogenetic cluster.

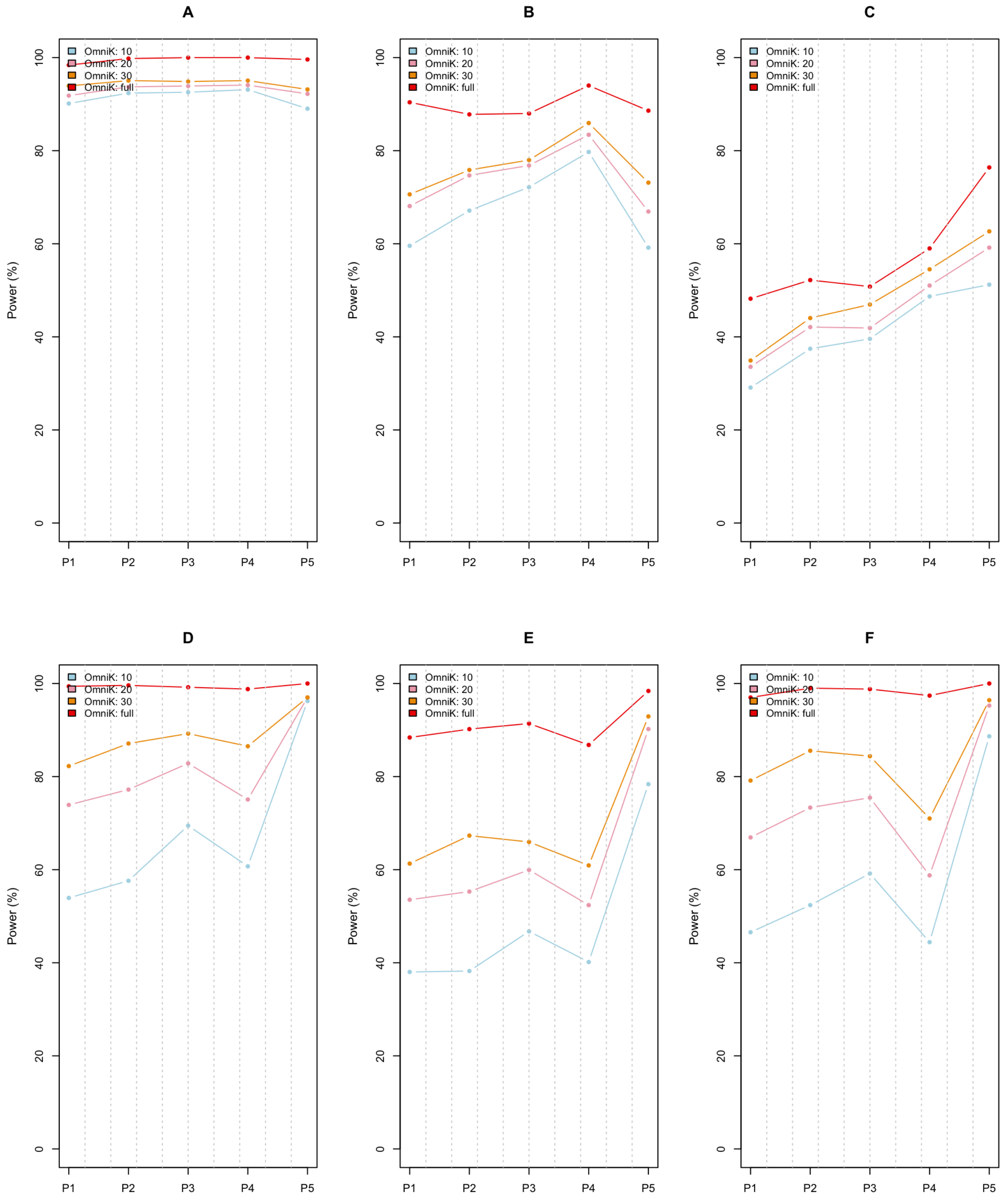

S2 Figure: Empirical powers for continuous response and randomized clinical trial ($n = 200$) using OmniK with 10 df, OmniK with 20 df, OmniK with 30 df and OmniK with full df. * The parameters of the Dirichlet-multinomial distribution were estimated using the Charlson et al's upper-respiratory-tract microbiome data. * A is for linear relationship with main effects; B is for linear relationship with interaction effects; C is for linear relationship with both of main and interaction effects; D is for nonlinear discrete relationship with main effects; E is for nonlinear discrete relationship with interaction effects; F is for nonlinear discrete relationship with both of main and interaction effects. * P1-P5 represents a selected phylogenetic cluster.

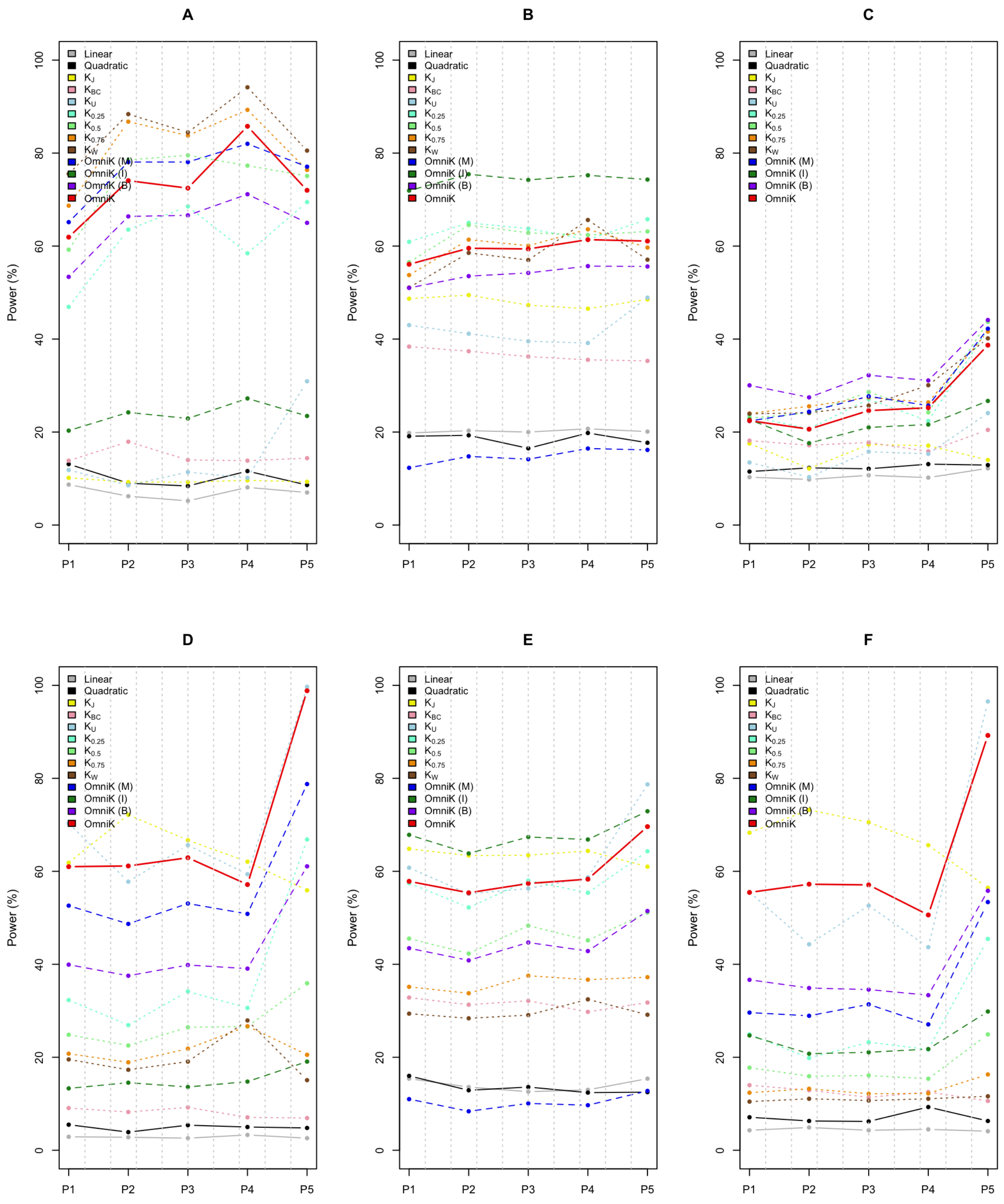

S3 Figure: Empirical powers for continuous response and observational study ($n = 100$) using (i) existing methods: CKAT based on linear and quadratic kernels, respectively (see Linear and Quadratic); (ii) general kernel machine regression analysis for each ecological kernel (see K_J, K_{BC}, K_U, $K_{0.25}$, $K_{0.5}$, $K_{0.75}$ and K_W); (iii) omnibus testing approach for each endogenous kernel on main effects, interaction effects or both of them (see OmniK (M), OmniK (I), OmniK (B)); and (iv) omnibus testing approach across all endogenous and input kernels (see OmniK). * The parameters of the Dirichlet-multinomial distribution were estimated using the Charlson et al's upper-respiratory-tract microbiome data.* A is for linear relationship with main effects; B is for linear relationship with interaction effects; C is for linear relationship with both of main and interaction effects; D is for nonlinear discrete relationship with main effects; E is for nonlinear discrete relationship with interaction effects; F is for nonlinear discrete relationship with both of main and interaction effects. * P1-P5 represents a selected phylogenetic cluster.

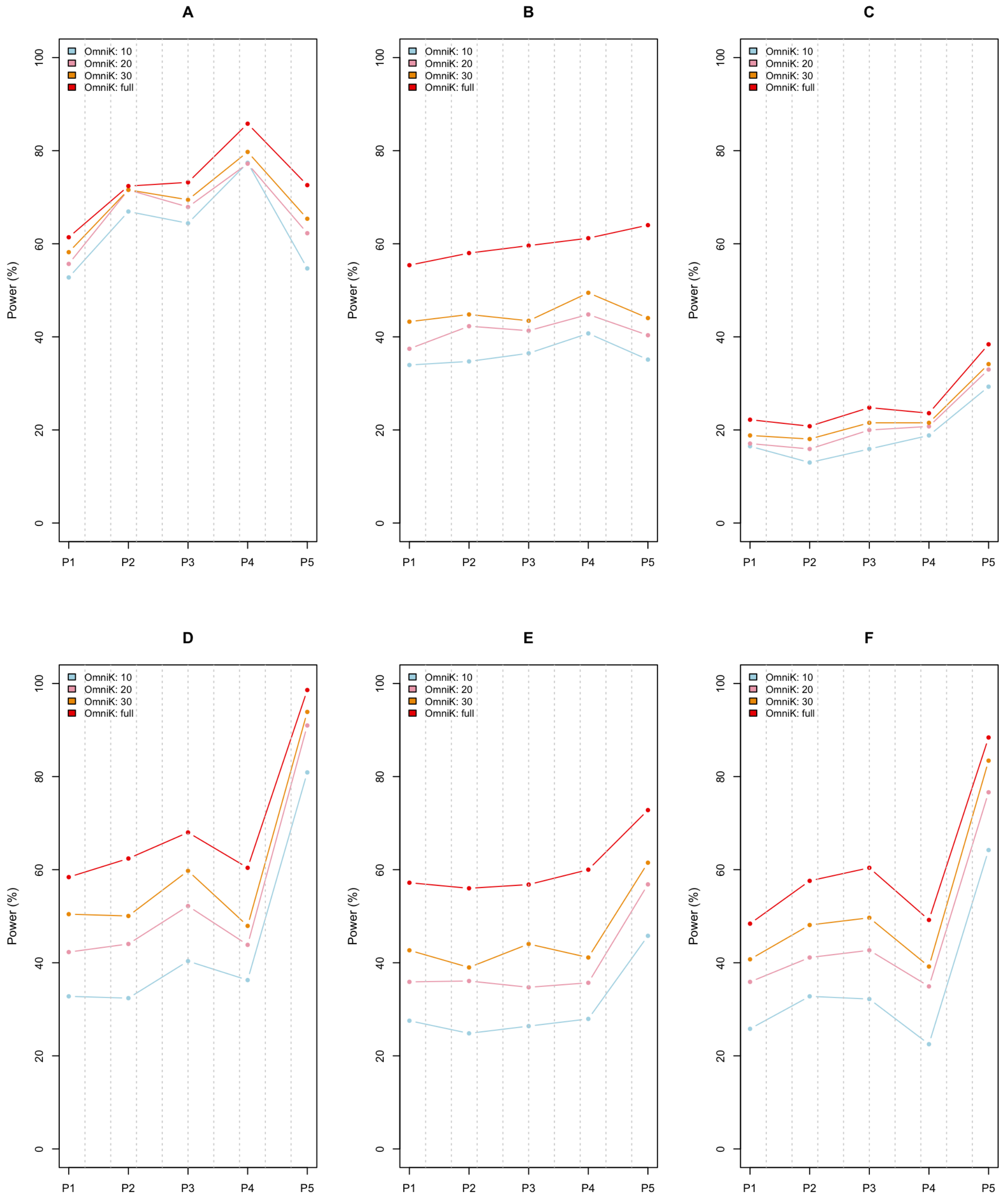

S4 Figure: Empirical powers for continuous response and observational study ($n = 100$) using OmniK with 10 df, OmniK with 20 df, OmniK with 30 df and OmniK with full df. * The parameters of the Dirichlet-multinomial distribution were estimated using the Charlson et al's upper-respiratory-tract microbiome data. * A is for linear relationship with main effects; B is for linear relationship with interaction effects; C is for linear relationship with both of main and interaction effects; D is for nonlinear discrete relationship with main effects; E is for nonlinear discrete relationship with interaction effects; F is for nonlinear discrete relationship with both of main and interaction effects. * P1-P5 represents a selected phylogenetic cluster.

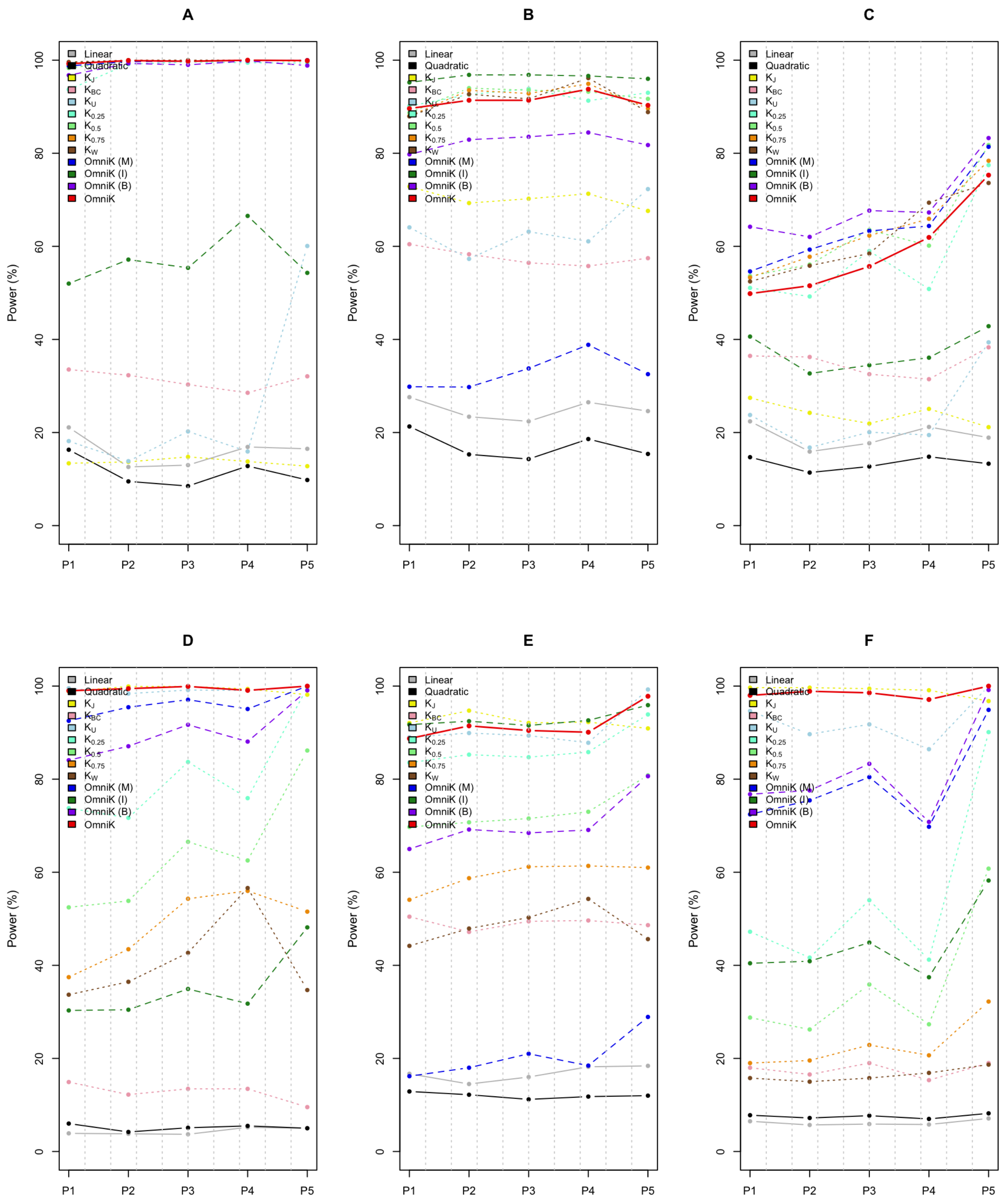

S5 Figure: Empirical powers for continuous response and observational study ($n = 200$) using (i) existing methods: CKAT based on linear and quadratic kernels, respectively (see Linear and Quadratic); (ii) general kernel machine regression analysis for each ecological kernel (see K_J, K_{BC}, K_U, $K_{0.25}$, $K_{0.5}$, $K_{0.75}$ and K_W); (iii) omnibus testing approach for each endogenous kernel on main effects, interaction effects or both of them (see OmniK (M), OmniK (I), OmniK (B)); and (iv) omnibus testing approach across all endogenous and input kernels (see OmniK). * The parameters of the Dirichlet-multinomial distribution were estimated using the Charlson et al's upper-respiratory-tract microbiome data.* A is for linear relationship with main effects; B is for linear relationship with interaction effects; C is for linear relationship with both of main and interaction effects; D is for nonlinear discrete relationship with main effects; E is for nonlinear discrete relationship with interaction effects; F is for nonlinear discrete relationship with both of main and interaction effects. * P1-P5 represents a selected phylogenetic cluster.

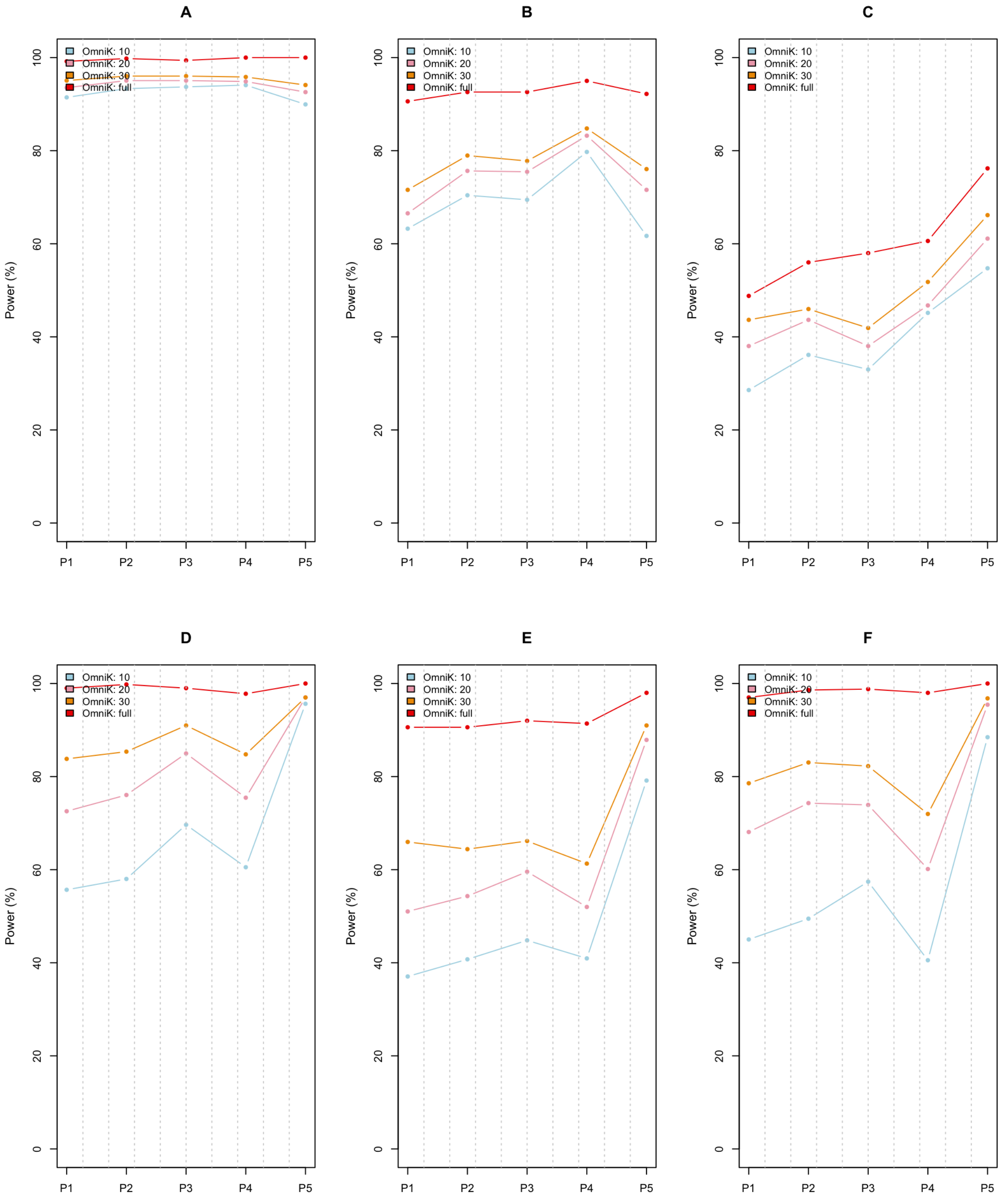

S6 Figure: Empirical powers for continuous response and observational study ($n = 200$) using OmniK with 10 df, OmniK with 20 df, OmniK with 30 df and OmniK with full df. * The parameters of the Dirichlet-multinomial distribution were estimated using the Charlson et al's upper-respiratory-tract microbiome data. * A is for linear relationship with main effects; B is for linear relationship with interaction effects; C is for linear relationship with both of main and interaction effects; D is for nonlinear discrete relationship with main effects; E is for nonlinear discrete relationship with interaction effects; F is for nonlinear discrete relationship with both of main and interaction effects. * P1-P5 represents a selected phylogenetic cluster.

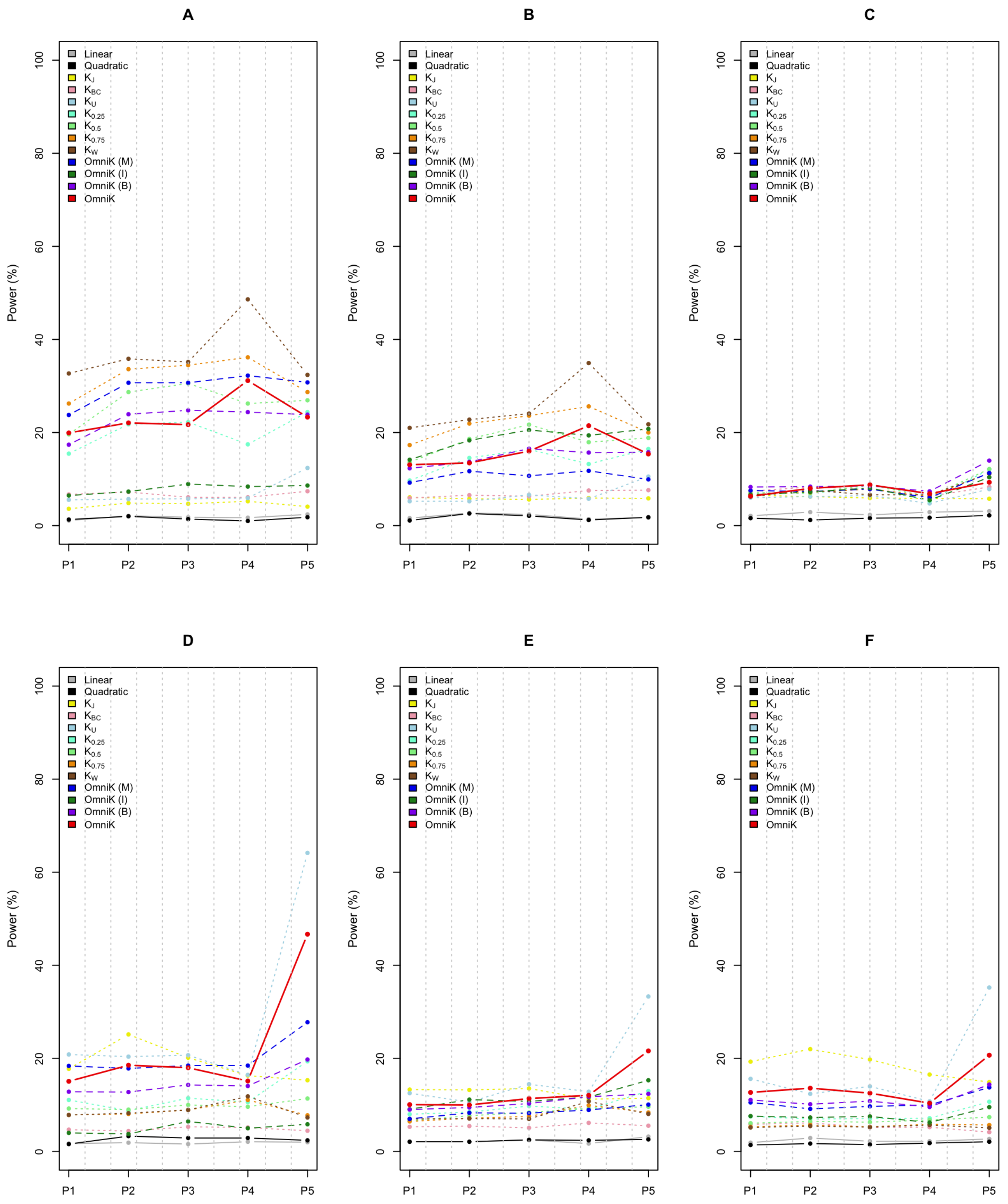

S7 Figure: Empirical powers for binary response and randomized clinical trial ($n = 100$) using (i) existing methods: CKAT based on linear and quadratic kernels, respectively (see Linear and Quadratic); (ii) general kernel machine regression analysis for each ecological kernel (see K_J, K_{BC}, K_U, $K_{0.25}$, $K_{0.5}$, $K_{0.75}$ and K_W); (iii) omnibus testing approach for each endogenous kernel on main effects, interaction effects or both of them (see OmniK (M), OmniK (I), OmniK (B)); and (iv) omnibus testing approach across all endogenous and input kernels (see OmniK). * The parameters of the Dirichlet-multinomial distribution were estimated using the Charlson et al's upper-respiratory-tract microbiome data.* A is for linear relationship with main effects; B is for linear relationship with interaction effects; C is for linear relationship with both of main and interaction effects; D is for nonlinear discrete relationship with main effects; E is for nonlinear discrete relationship with interaction effects; F is for nonlinear discrete relationship with both of main and interaction effects. * P1-P5 represents a selected phylogenetic cluster.

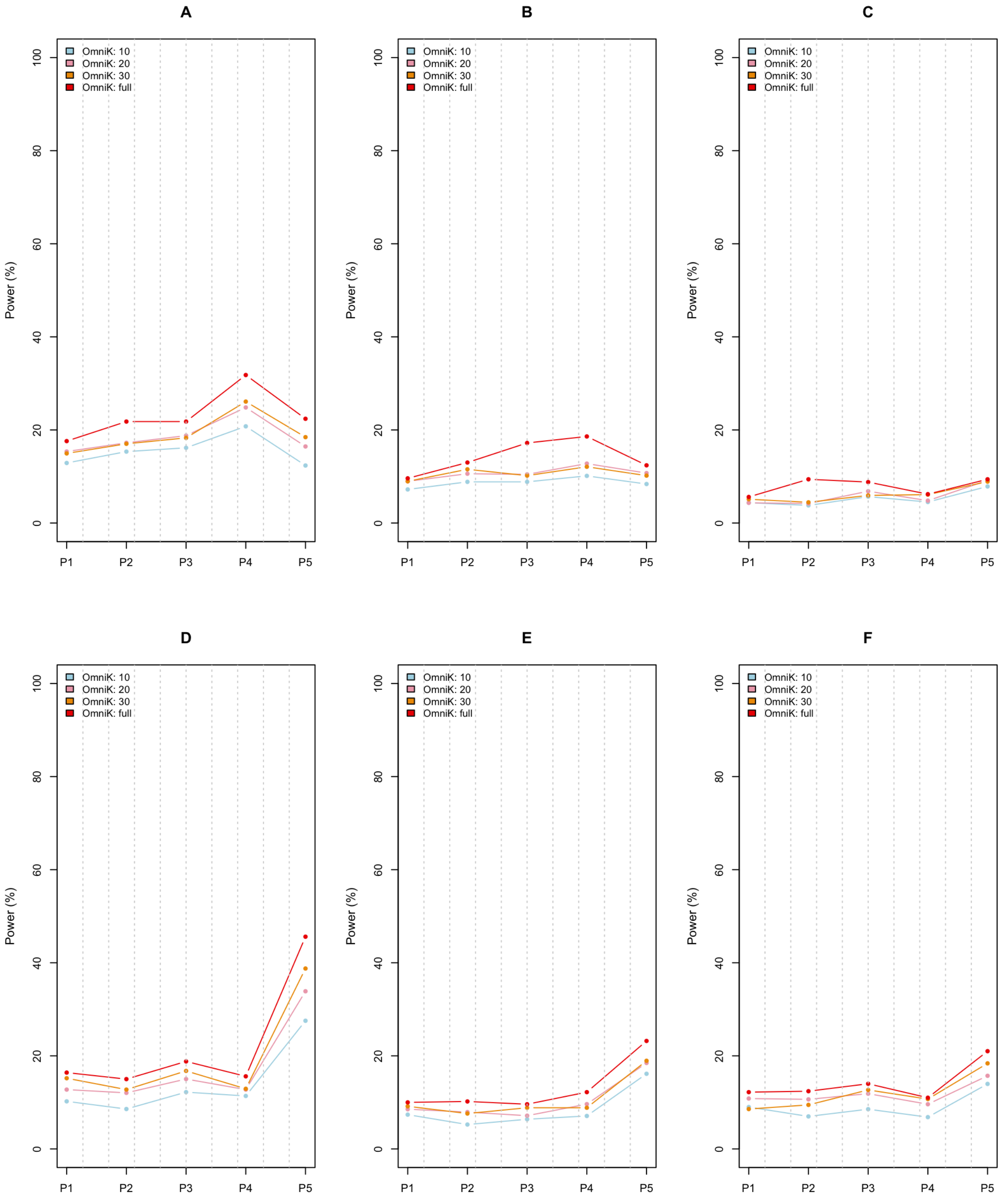

S8 Figure: Empirical powers for binary response and randomized clinical trial ($n = 100$) using OmniK with 10 df, OmniK with 20 df, OmniK with 30 df and OmniK with full df. * The parameters of the Dirichlet-multinomial distribution were estimated using the Charlson et al's upper-respiratory-tract microbiome data. * A is for linear relationship with main effects; B is for linear relationship with interaction effects; C is for linear relationship with both of main and interaction effects; D is for nonlinear discrete relationship with main effects; E is for nonlinear discrete relationship with interaction effects; F is for nonlinear discrete relationship with both of main and interaction effects. * P1-P5 represents a selected phylogenetic cluster.

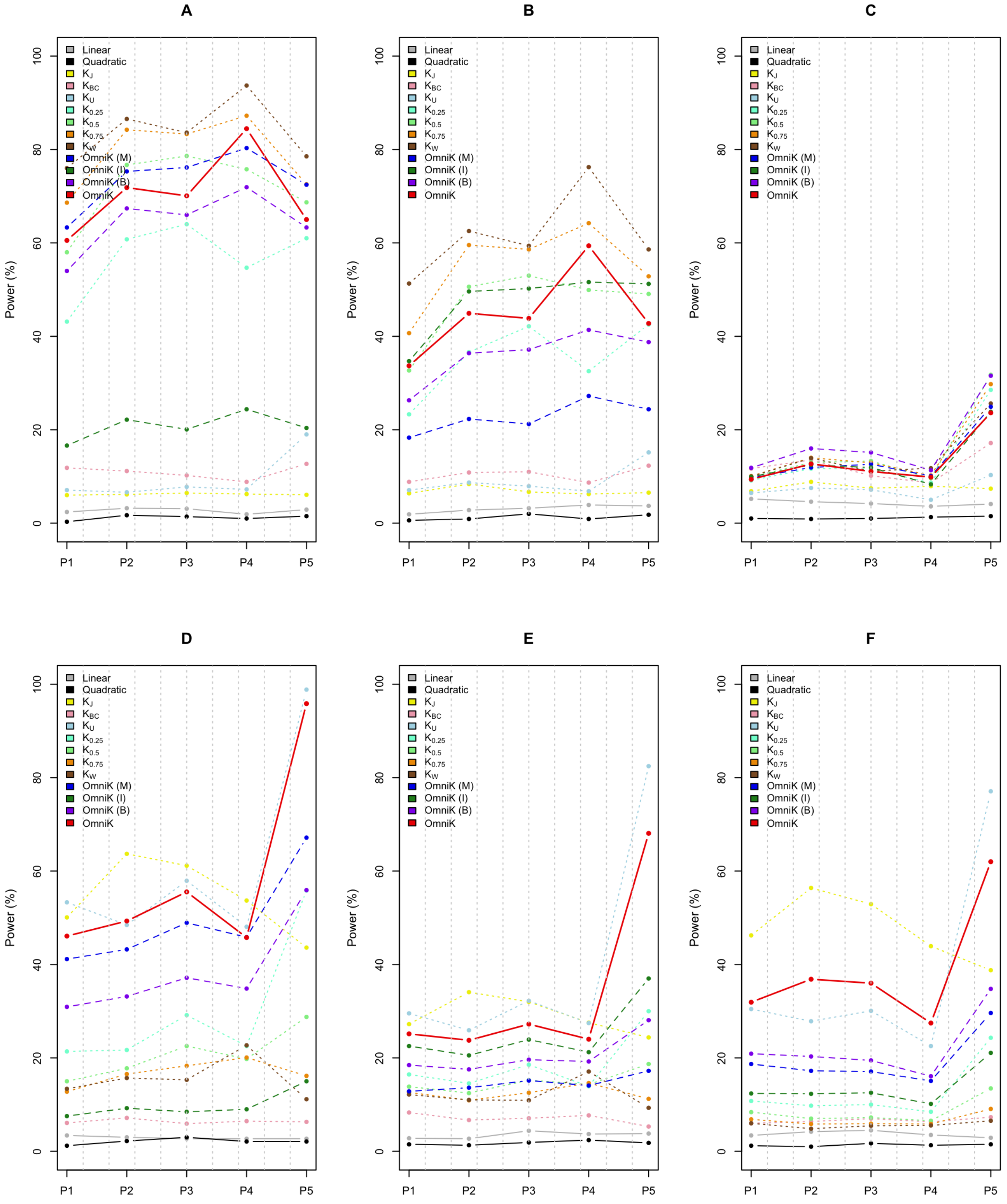

S9 Figure: Empirical powers for binary response and randomized clinical trial ($n = 200$) using (i) existing methods: CKAT based on linear and quadratic kernels, respectively (see Linear and Quadratic); (ii) general kernel machine regression analysis for each ecological kernel (see K_J, K_{BC}, K_U, $K_{0.25}$, $K_{0.5}$, $K_{0.75}$ and K_W); (iii) omnibus testing approach for each endogenous kernel on main effects, interaction effects or both of them (see OmniK (M), OmniK (I), OmniK (B)); and (iv) omnibus testing approach across all endogenous and input kernels (see OmniK). * The parameters of the Dirichlet-multinomial distribution were estimated using the Charlson et al's upper-respiratory-tract microbiome data.* A is for linear relationship with main effects; B is for linear relationship with interaction effects; C is for linear relationship with both of main and interaction effects; D is for nonlinear discrete relationship with main effects; E is for nonlinear discrete relationship with interaction effects; F is for nonlinear discrete relationship with both of main and interaction effects. * P1-P5 represents a selected phylogenetic cluster.

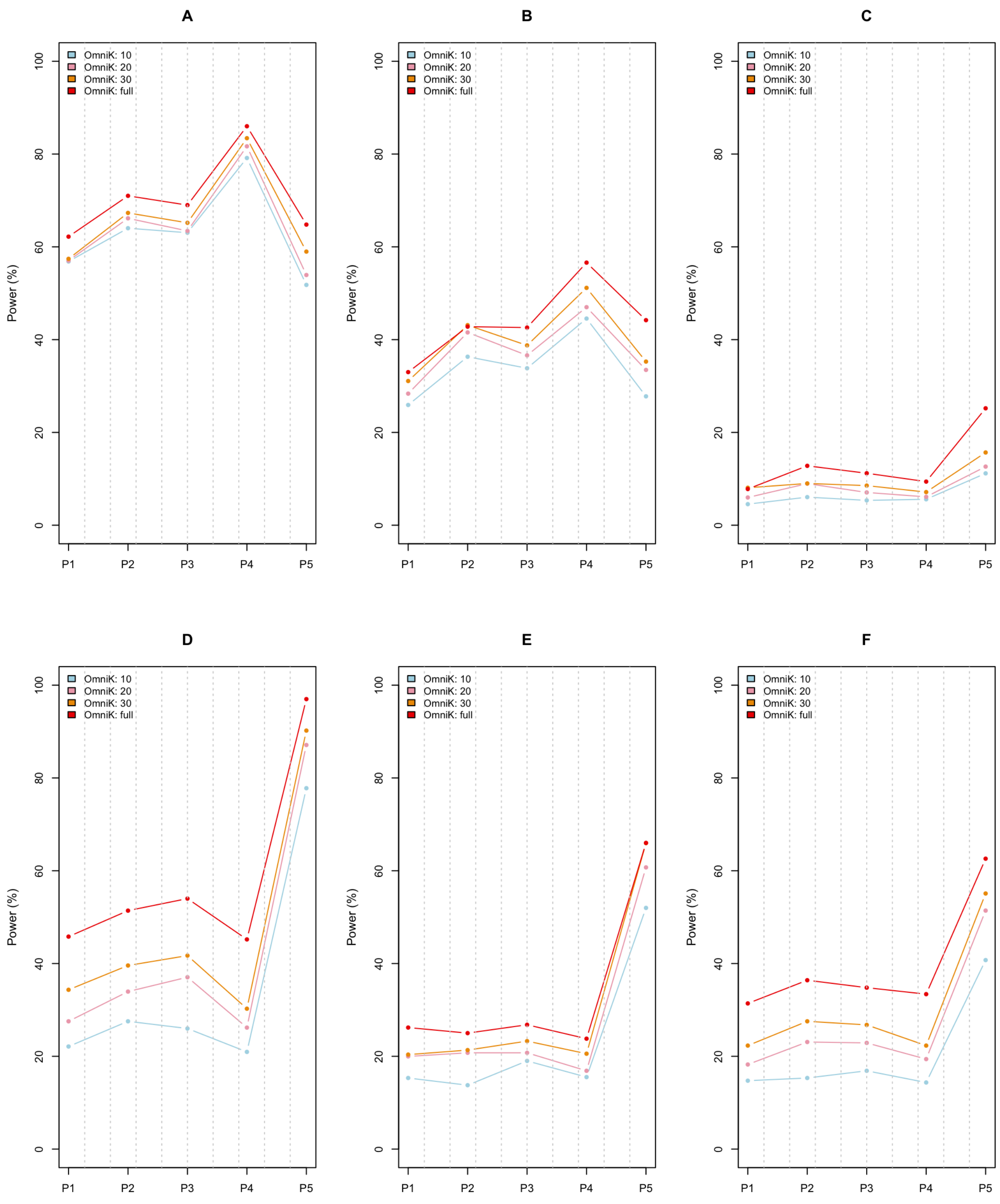

S10 Figure: Empirical powers for binary response and randomized clinical trial ($n = 200$) using OmniK with 10 df, OmniK with 20 df, OmniK with 30 df and OmniK with full df. * The parameters of the Dirichlet-multinomial distribution were estimated using the Charlson et al's upper-respiratory-tract microbiome data. * A is for linear relationship with main effects; B is for linear relationship with interaction effects; C is for linear relationship with both of main and interaction effects; D is for nonlinear discrete relationship with main effects; E is for nonlinear discrete relationship with interaction effects; F is for nonlinear discrete relationship with both of main and interaction effects. * P1-P5 represents a selected phylogenetic cluster.

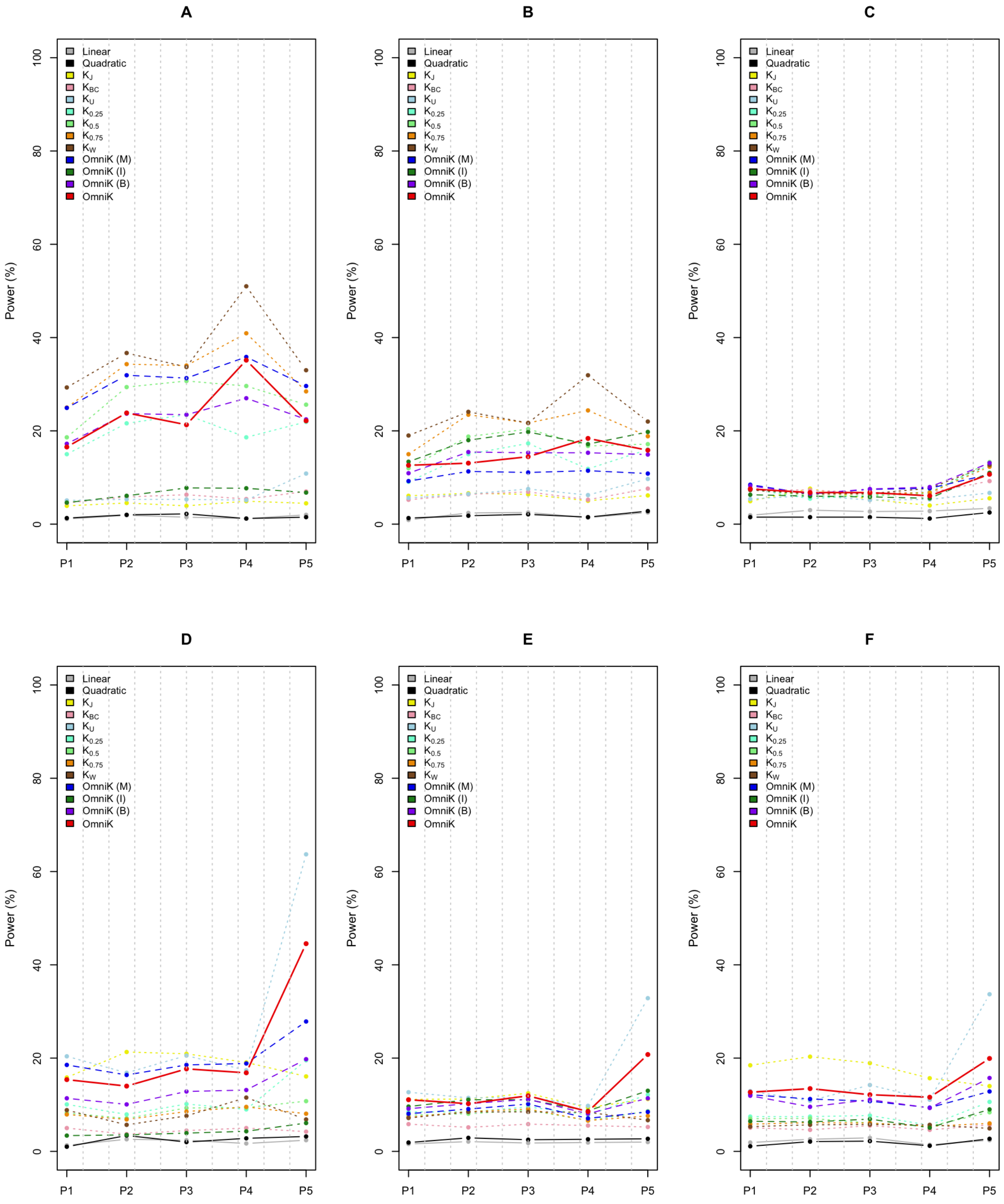

S11 Figure: Empirical powers for binary response and observational study ($n = 100$) using (i) existing methods: CKAT based on linear and quadratic kernels, respectively (see Linear and Quadratic); (ii) general kernel machine regression analysis for each ecological kernel (see K_J, K_{BC}, K_U, $K_{0.25}$, $K_{0.5}$, $K_{0.75}$ and K_W); (iii) omnibus testing approach for each endogenous kernel on main effects, interaction effects or both of them (see OmniK (M), OmniK (I), OmniK (B)); and (iv) omnibus testing approach across all endogenous and input kernels (see OmniK). * The parameters of the Dirichlet-multinomial distribution were estimated using the Charlson et al's upper-respiratory-tract microbiome data.* A is for linear relationship with main effects; B is for linear relationship with interaction effects; C is for linear relationship with both of main and interaction effects; D is for nonlinear discrete relationship with main effects; E is for nonlinear discrete relationship with interaction effects; F is for nonlinear discrete relationship with both of main and interaction effects. * P1-P5 represents a selected phylogenetic cluster.

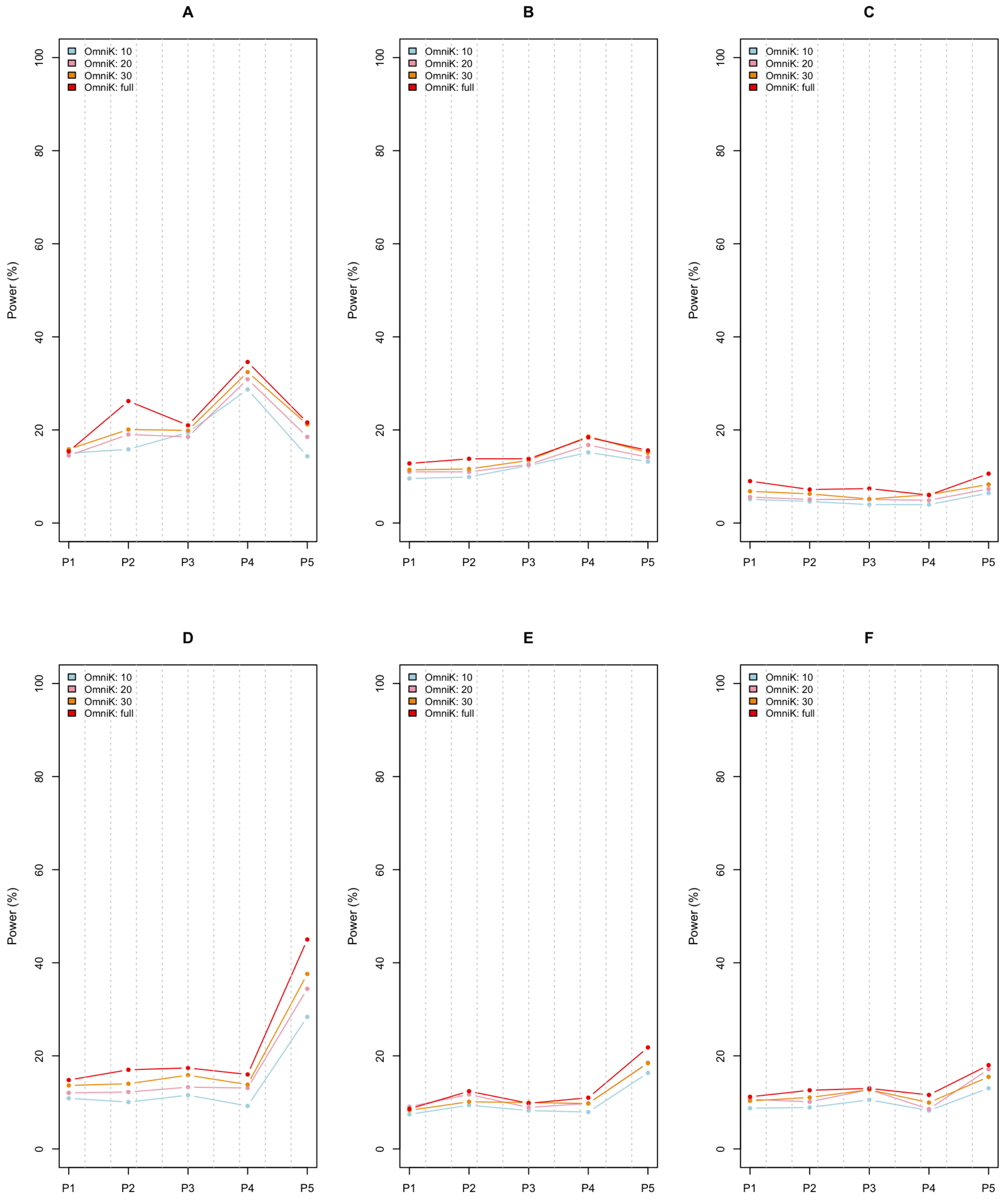

S12 Figure: Empirical powers for binary response and observational study ($n = 100$) using OmniK with 10 df, OmniK with 20 df, OmniK with 30 df and OmniK with full df. * The parameters of the Dirichlet-multinomial distribution were estimated using the Charlson et al's upper-respiratory-tract microbiome data. * A is for linear relationship with main effects; B is for linear relationship with interaction effects; C is for linear relationship with both of main and interaction effects; D is for nonlinear discrete relationship with main effects; E is for nonlinear discrete relationship with interaction effects; F is for nonlinear discrete relationship with both of main and interaction effects. * P1-P5 represents a selected phylogenetic cluster.

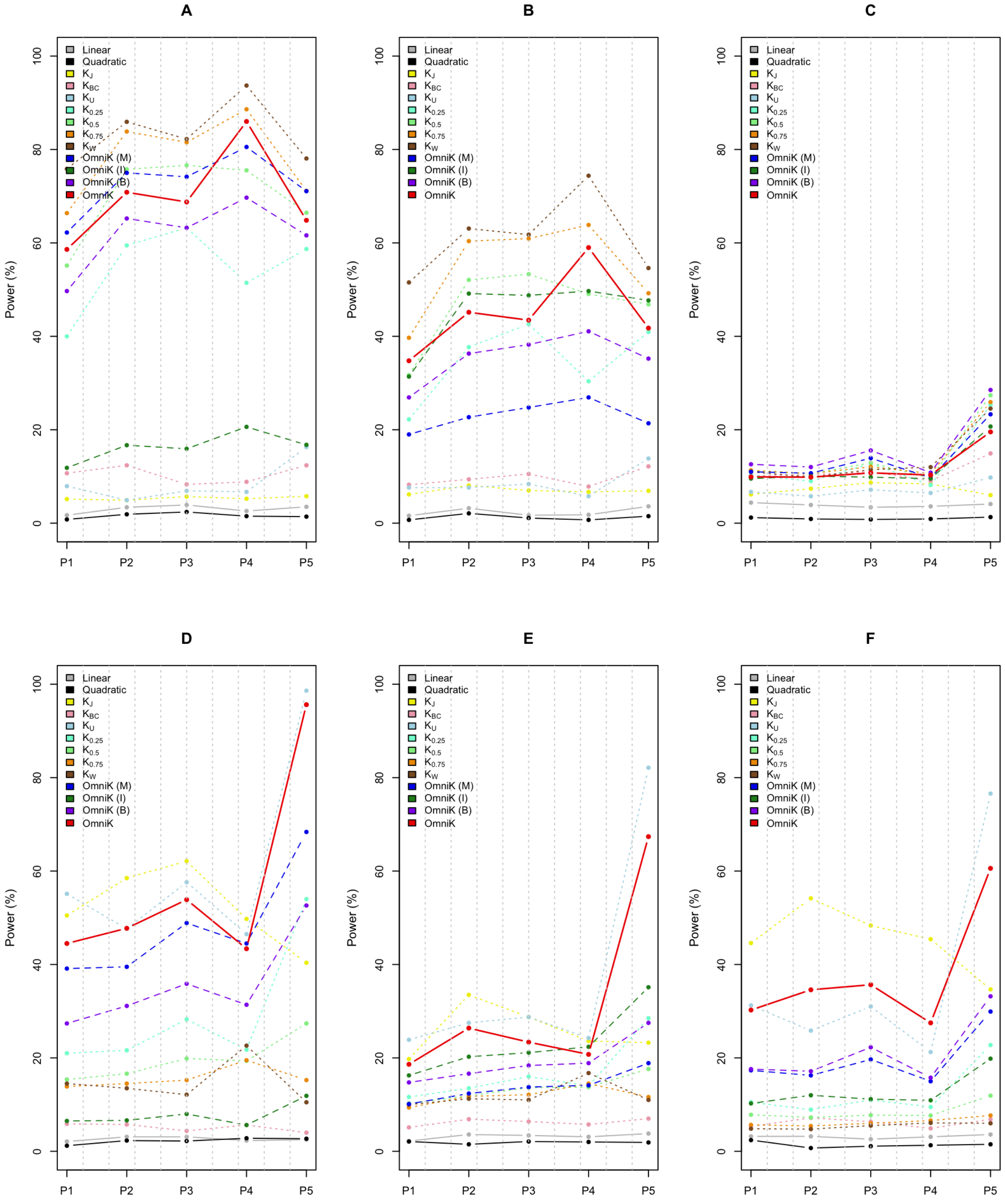

S13 Figure: Empirical powers for binary response and observational study ($n = 200$) using (i) existing methods: CKAT based on linear and quadratic kernels, respectively (see Linear and Quadratic); (ii) general kernel machine regression analysis for each ecological kernel (see K_J, K_{BC}, K_U, $K_{0.25}$, $K_{0.5}$, $K_{0.75}$ and K_W); (iii) omnibus testing approach for each endogenous kernel on main effects, interaction effects or both of them (see OmniK (M), OmniK (I), OmniK (B)); and (iv) omnibus testing approach across all endogenous and input kernels (see OmniK). * The parameters of the Dirichlet-multinomial distribution were estimated using the Charlson et al's upper-respiratory-tract microbiome data.* A is for linear relationship with main effects; B is for linear relationship with interaction effects; C is for linear relationship with both of main and interaction effects; D is for nonlinear discrete relationship with main effects; E is for nonlinear discrete relationship with interaction effects; F is for nonlinear discrete relationship with both of main and interaction effects. * P1-P5 represents a selected phylogenetic cluster.

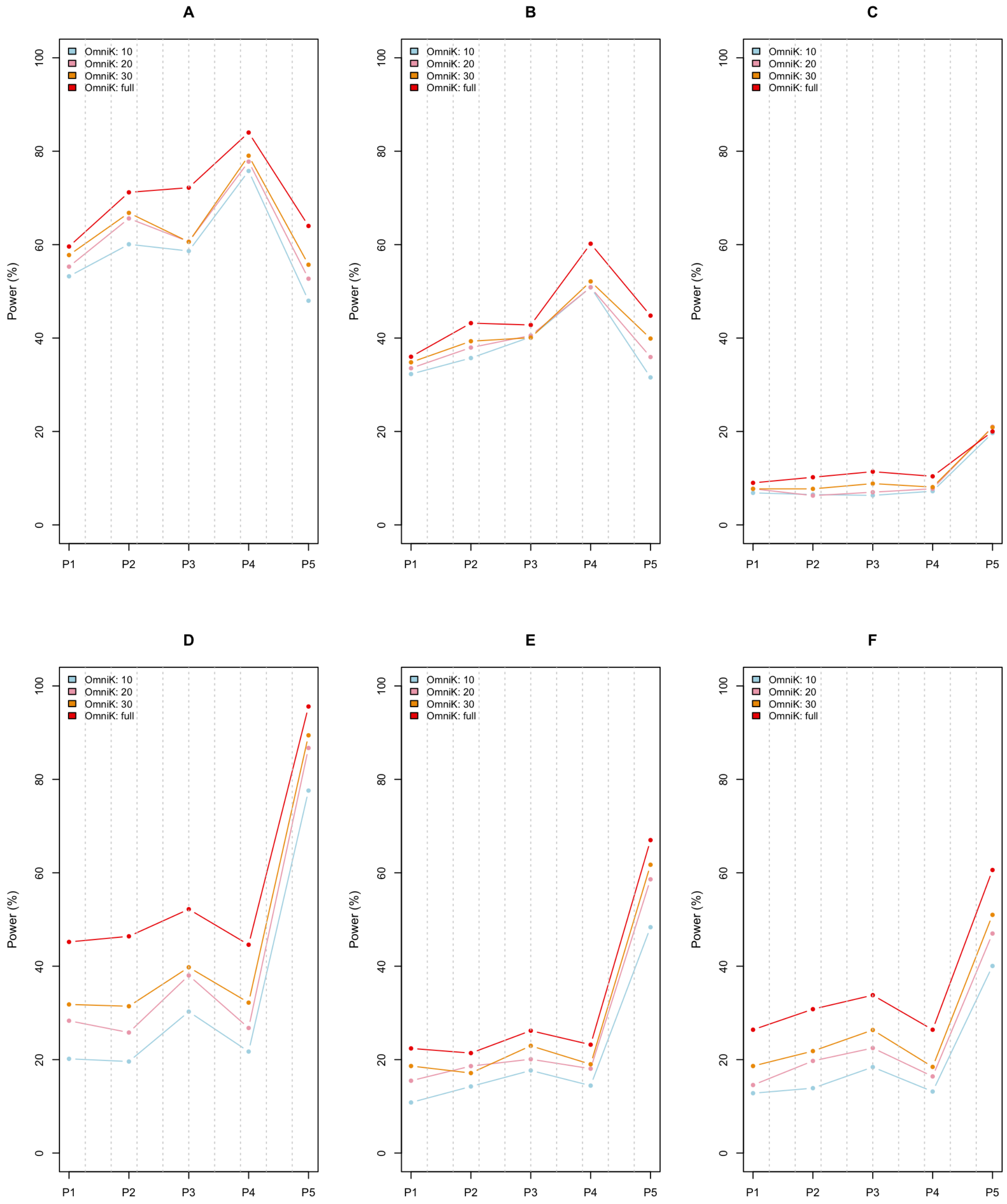

S14 Figure: Empirical powers for binary response and observational study ($n = 200$) using OmniK with 10 df, OmniK with 20 df, OmniK with 30 df and OmniK with full df. * The parameters of the Dirichlet-multinomial distribution were estimated using the Charlson et al's upper-respiratory-tract microbiome data. * A is for linear relationship with main effects; B is for linear relationship with interaction effects; C is for linear relationship with both of main and interaction effects; D is for nonlinear discrete relationship with main effects; E is for nonlinear discrete relationship with interaction effects; F is for nonlinear discrete relationship with both of main and interaction effects. * P1-P5 represents a selected phylogenetic cluster.

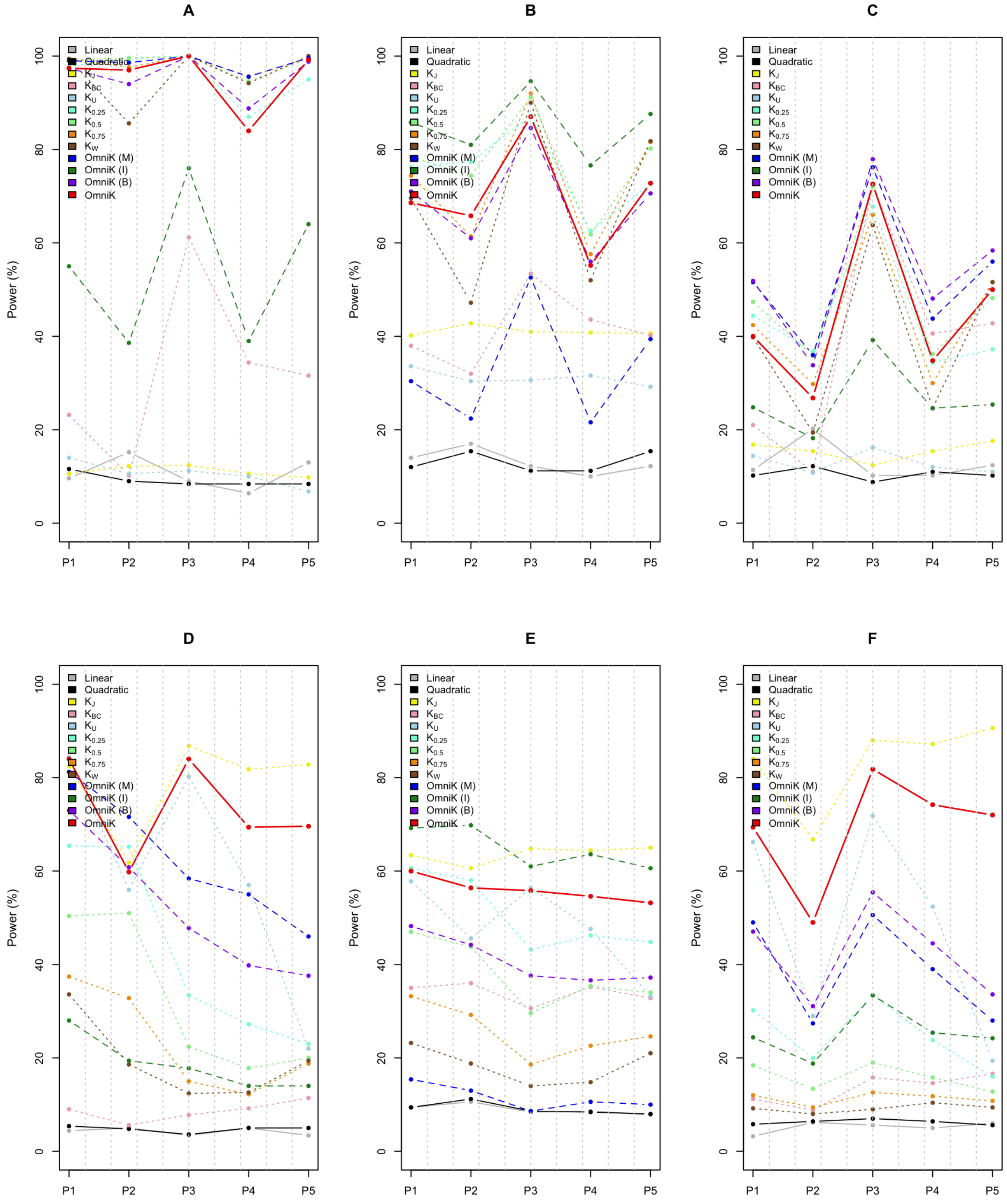

S15 Figure: Empirical powers for continuous response and randomized clinical trial ($n = 100$) using (i) existing methods: CKAT based on linear and quadratic kernels, respectively (see Linear and Quadratic); (ii) general kernel machine regression analysis for each ecological kernel (see K_J, K_{BC}, K_U, $K_{0.25}$, $K_{0.5}$, $K_{0.75}$ and K_W); (iii) omnibus testing approach for each endogenous kernel on main effects, interaction effects or both of them (see OmniK (M), OmniK (I), OmniK (B)); and (iv) omnibus testing approach across all endogenous and input kernels (see OmniK). * The parameters of the Dirichlet-multinomial distribution were estimated using the Yanai et al's gut microbiome data.* A is for linear relationship with main effects; B is for linear relationship with interaction effects; C is for linear relationship with both of main and interaction effects; D is for nonlinear discrete relationship with main effects; E is for nonlinear discrete relationship with interaction effects; F is for nonlinear discrete relationship with both of main and interaction effects. * P1-P5 represents a selected phylogenetic cluster.

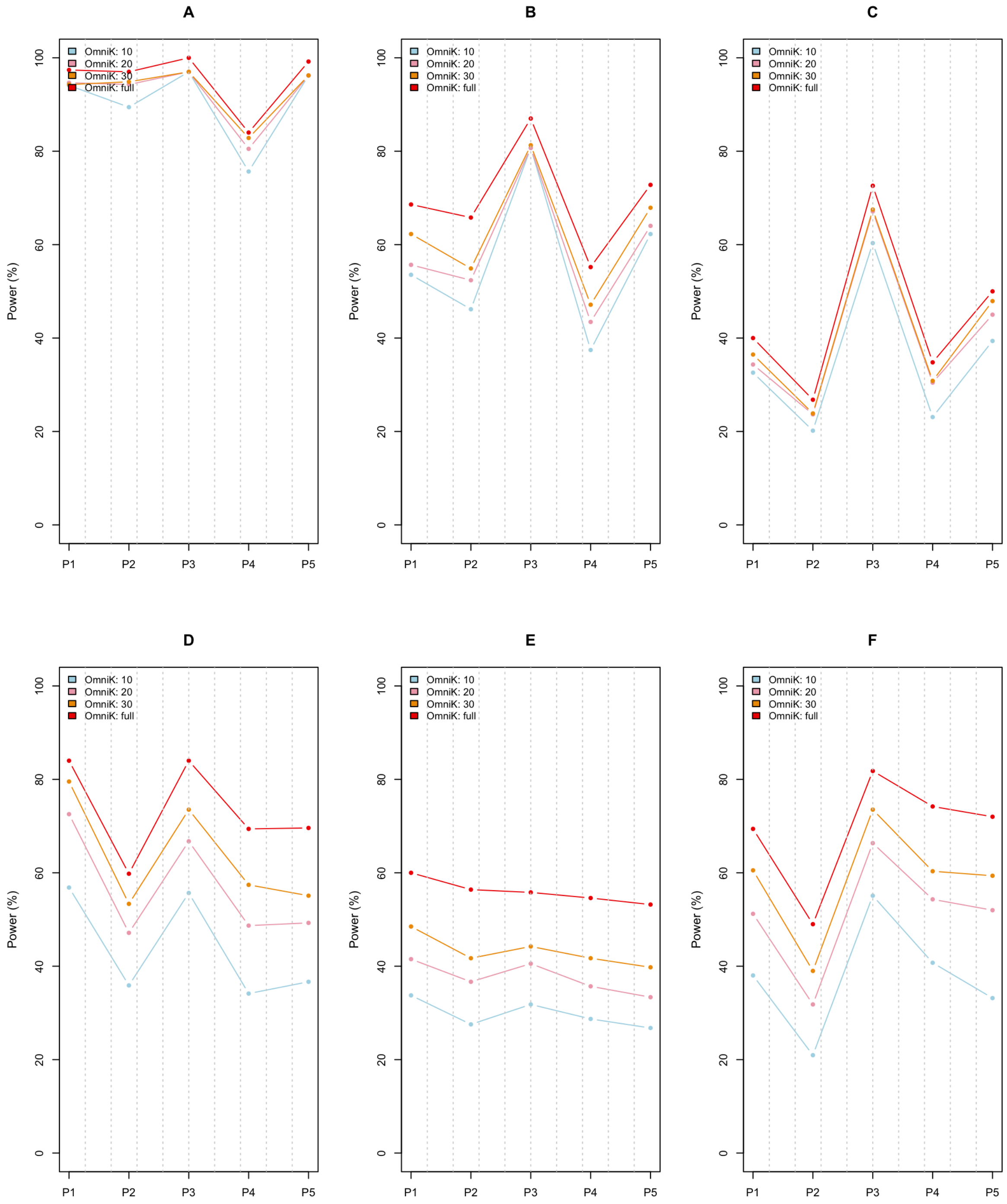

S16 Figure: Empirical powers for continuous response and randomized clinical trial ($n = 100$) using OmniK with 10 df, OmniK with 20 df, OmniK with 30 df and OmniK with full df. * The parameters of the Dirichlet-multinomial distribution were estimated using the Yanai et al's gut microbiome data. * A is for linear relationship with main effects; B is for linear relationship with interaction effects; C is for linear relationship with both of main and interaction effects; D is for nonlinear discrete relationship with main effects; E is for nonlinear discrete relationship with interaction effects; F is for nonlinear discrete relationship with both of main and interaction effects. * P1-P5 represents a selected phylogenetic cluster.

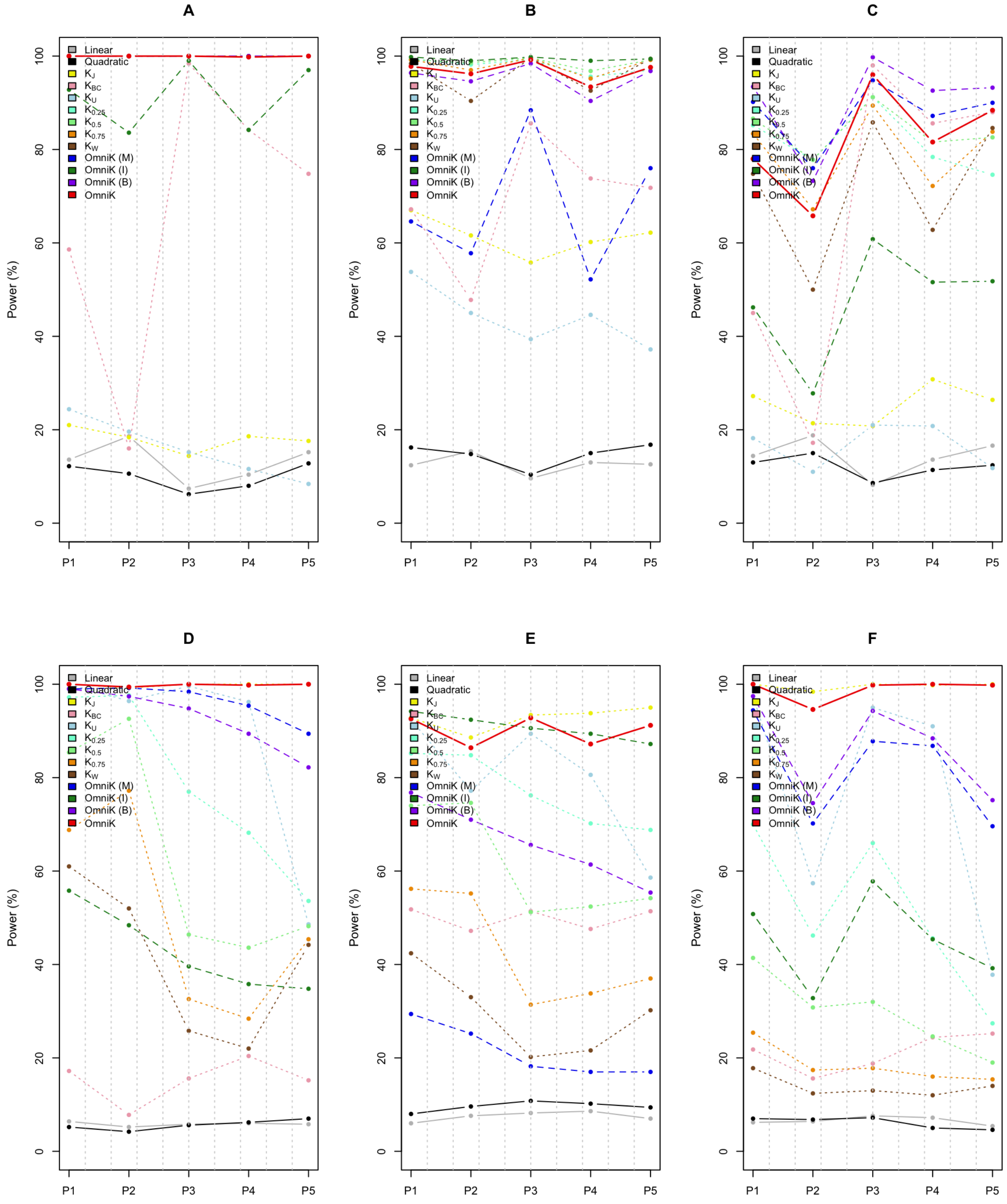

S17 Figure: Empirical powers for continuous response and randomized clinical trial ($n = 200$) using (i) existing methods: CKAT based on linear and quadratic kernels, respectively (see Linear and Quadratic); (ii) general kernel machine regression analysis for each ecological kernel (see K_J, K_{BC}, K_U, $K_{0.25}$, $K_{0.5}$, $K_{0.75}$ and K_W); (iii) omnibus testing approach for each endogenous kernel on main effects, interaction effects or both of them (see OmniK (M), OmniK (I), OmniK (B)); and (iv) omnibus testing approach across all endogenous and input kernels (see OmniK). * The parameters of the Dirichlet-multinomial distribution were estimated using the Yanai et al's gut microbiome data.* A is for linear relationship with main effects; B is for linear relationship with interaction effects; C is for linear relationship with both of main and interaction effects; D is for nonlinear discrete relationship with main effects; E is for nonlinear discrete relationship with interaction effects; F is for nonlinear discrete relationship with both of main and interaction effects. * P1-P5 represents a selected phylogenetic cluster.

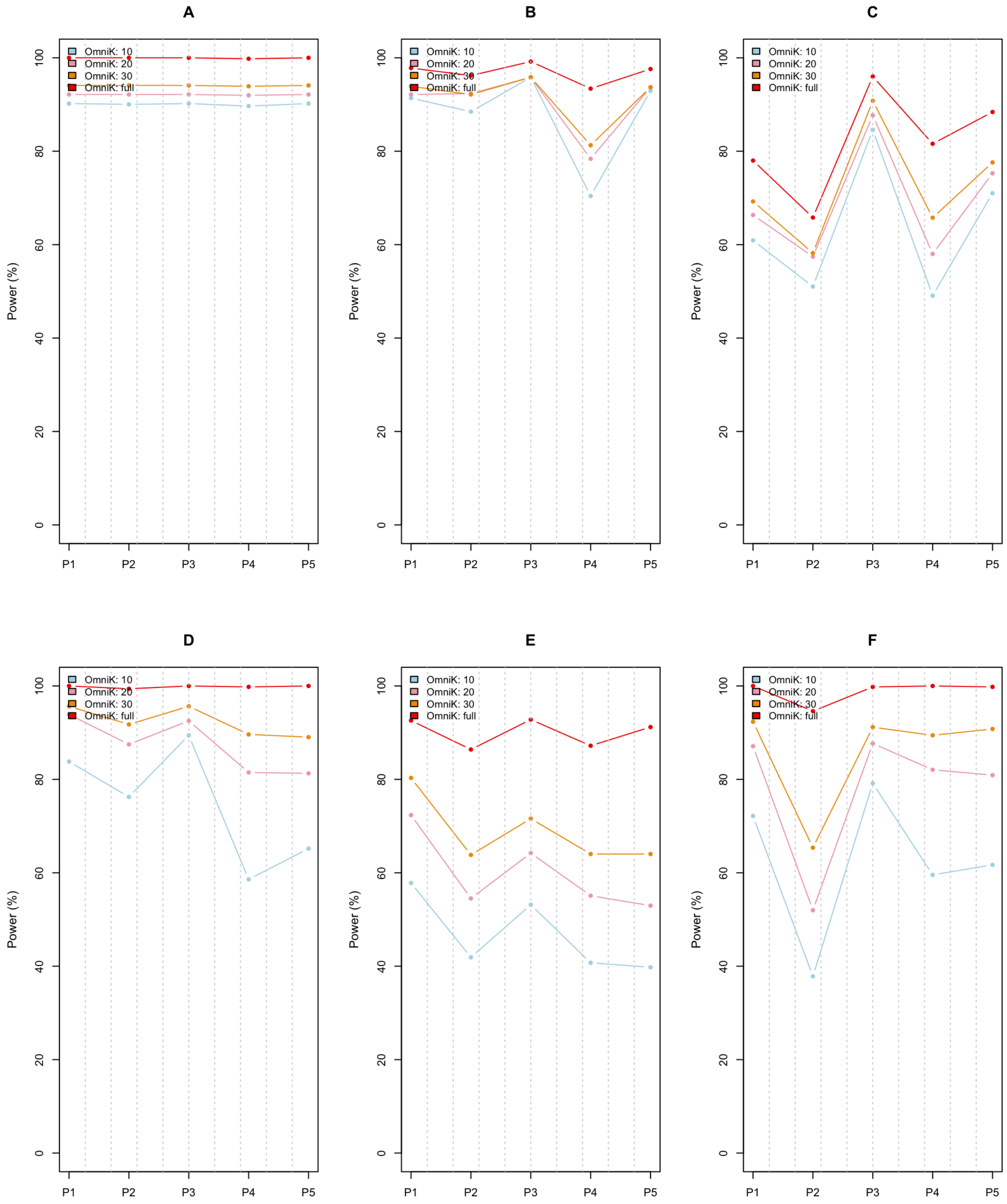

S18 Figure: Empirical powers for continuous response and randomized clinical trial ($n = 200$) using OmniK with 10 df, OmniK with 20 df, OmniK with 30 df and OmniK with full df. * The parameters of the Dirichlet-multinomial distribution were estimated using the Yanai et al's gut microbiome data. * A is for linear relationship with main effects; B is for linear relationship with interaction effects; C is for linear relationship with both of main and interaction effects; D is for nonlinear discrete relationship with main effects; E is for nonlinear discrete relationship with interaction effects; F is for nonlinear discrete relationship with both of main and interaction effects. * P1-P5 represents a selected phylogenetic cluster.

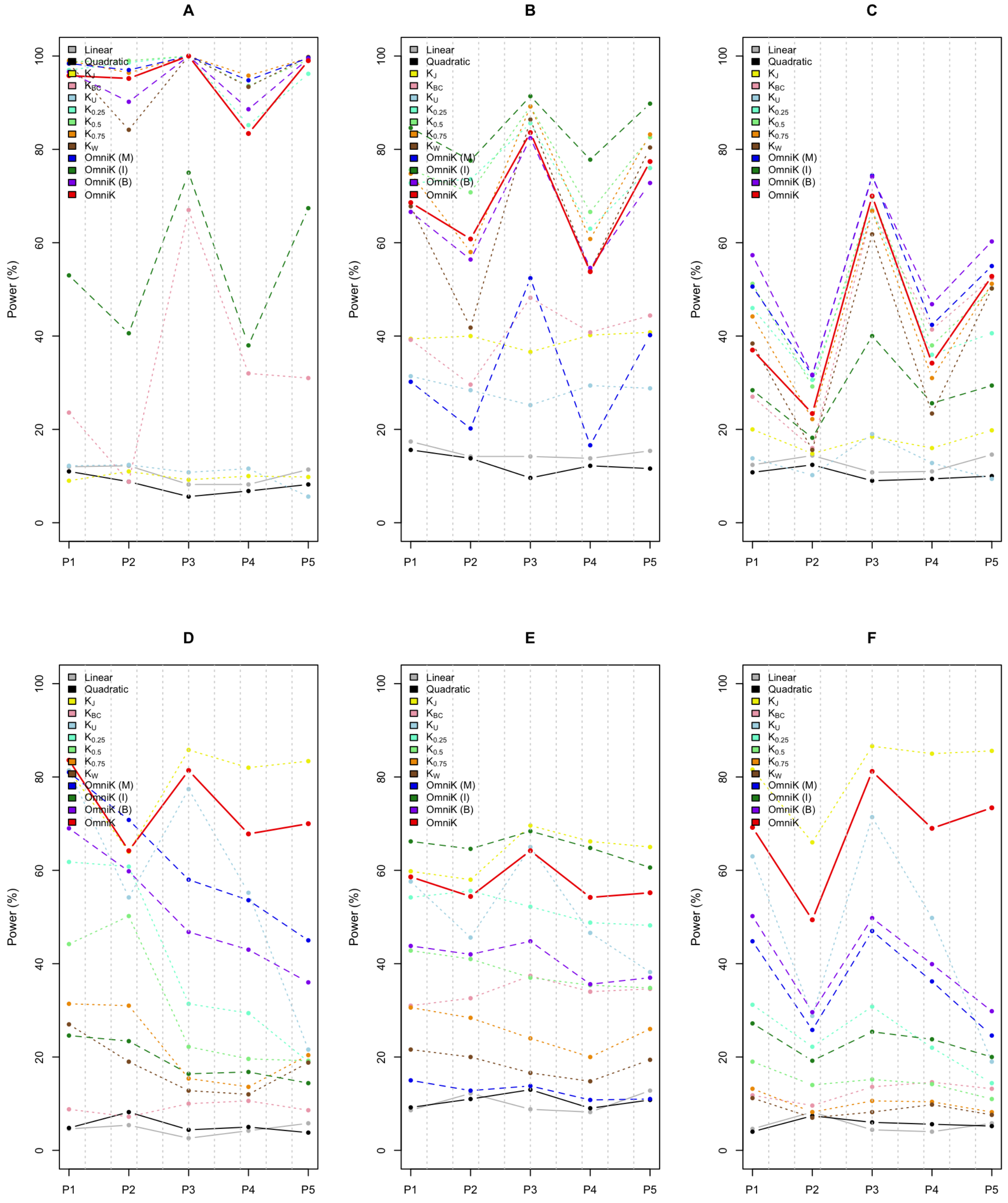

S19 Figure: Empirical powers for continuous response and observational study ($n = 100$) using (i) existing methods: CKAT based on linear and quadratic kernels, respectively (see Linear and Quadratic); (ii) general kernel machine regression analysis for each ecological kernel (see K_J, K_{BC}, K_U, $K_{0.25}$, $K_{0.5}$, $K_{0.75}$ and K_W); (iii) omnibus testing approach for each endogenous kernel on main effects, interaction effects or both of them (see OmniK (M), OmniK (I), OmniK (B)); and (iv) omnibus testing approach across all endogenous and input kernels (see OmniK). * The parameters of the Dirichlet-multinomial distribution were estimated using the Yanai et al's gut microbiome data.* A is for linear relationship with main effects; B is for linear relationship with interaction effects; C is for linear relationship with both of main and interaction effects; D is for nonlinear discrete relationship with main effects; E is for nonlinear discrete relationship with interaction effects; F is for nonlinear discrete relationship with both of main and interaction effects. * P1-P5 represents a selected phylogenetic cluster.

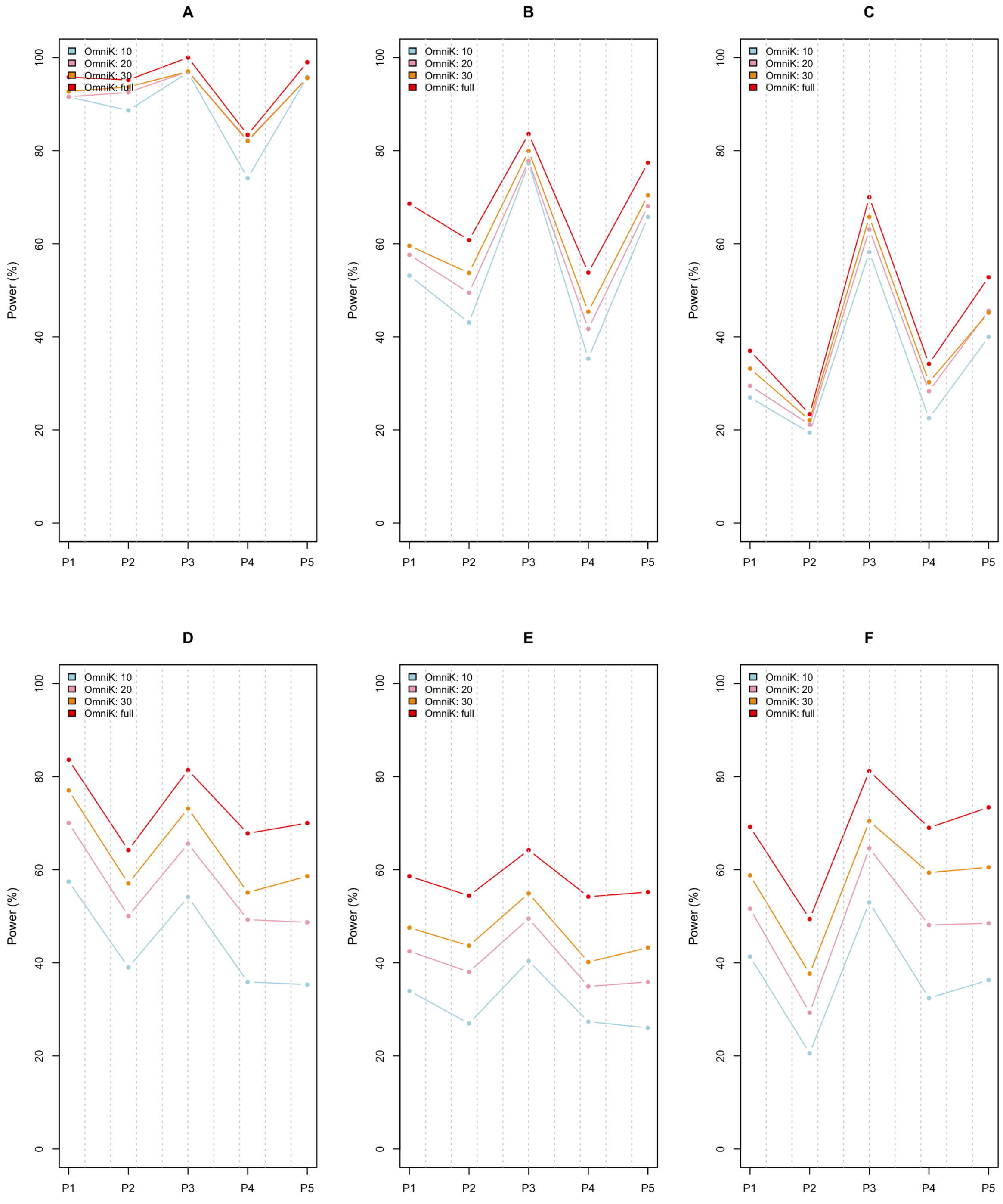

S20 Figure: Empirical powers for continuous response and observational study ($n = 100$) using OmniK with 10 df, OmniK with 20 df, OmniK with 30 df and OmniK with full df. * The parameters of the Dirichlet-multinomial distribution were estimated using the Yanai et al's gut microbiome data. * A is for linear relationship with main effects; B is for linear relationship with interaction effects; C is for linear relationship with both of main and interaction effects; D is for nonlinear discrete relationship with main effects; E is for nonlinear discrete relationship with interaction effects; F is for nonlinear discrete relationship with both of main and interaction effects. * P1-P5 represents a selected phylogenetic cluster.

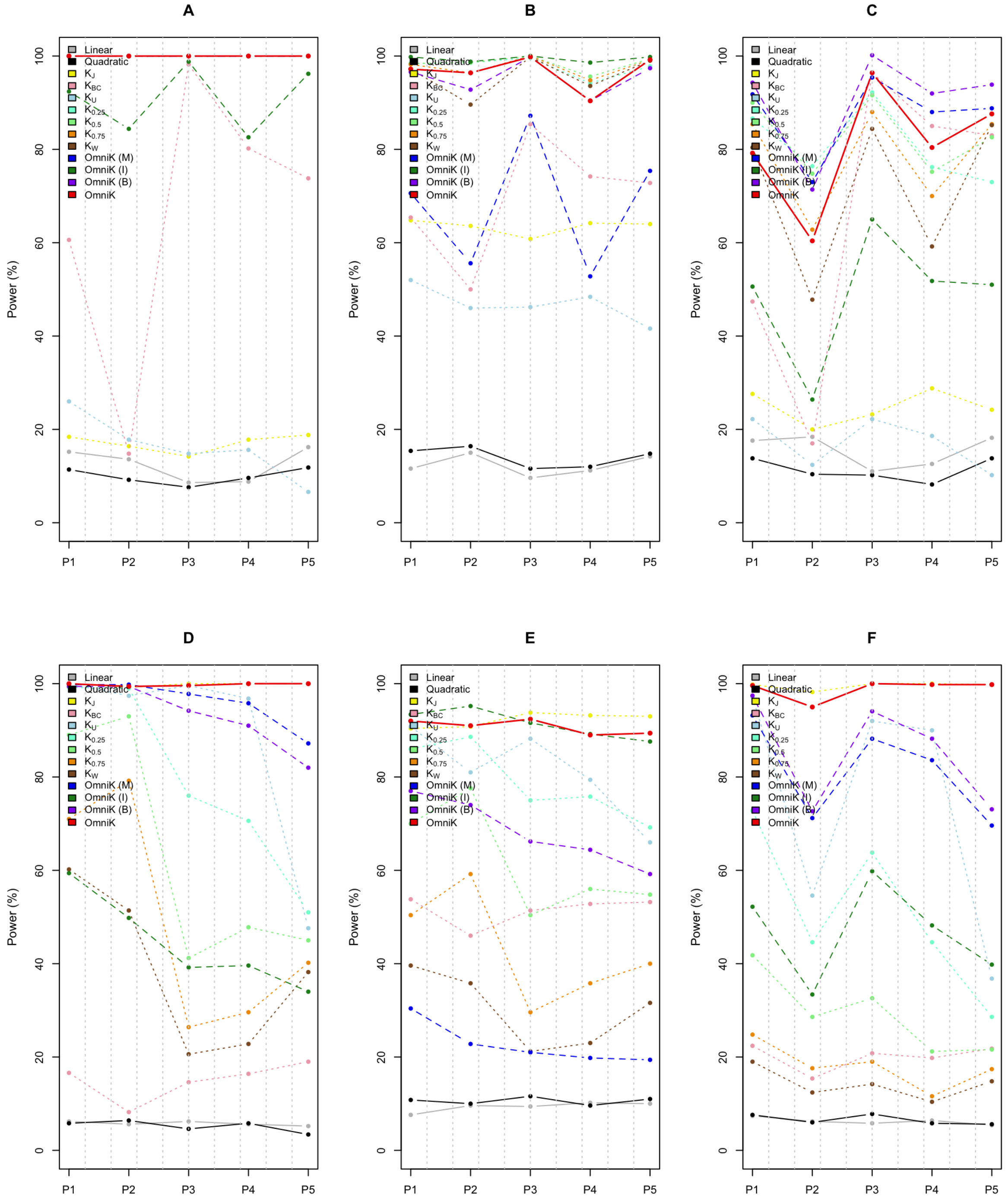

S21 Figure: Empirical powers for continuous response and observational study ($n = 200$) using (i) existing methods: CKAT based on linear and quadratic kernels, respectively (see Linear and Quadratic); (ii) general kernel machine regression analysis for each ecological kernel (see K_J, K_{BC}, K_U, $K_{0.25}$, $K_{0.5}$, $K_{0.75}$ and K_W); (iii) omnibus testing approach for each endogenous kernel on main effects, interaction effects or both of them (see OmniK (M), OmniK (I), OmniK (B)); and (iv) omnibus testing approach across all endogenous and input kernels (see OmniK). * The parameters of the Dirichlet-multinomial distribution were estimated using the Yanai et al's gut microbiome data.* A is for linear relationship with main effects; B is for linear relationship with interaction effects; C is for linear relationship with both of main and interaction effects; D is for nonlinear discrete relationship with main effects; E is for nonlinear discrete relationship with interaction effects; F is for nonlinear discrete relationship with both of main and interaction effects. * P1-P5 represents a selected phylogenetic cluster.

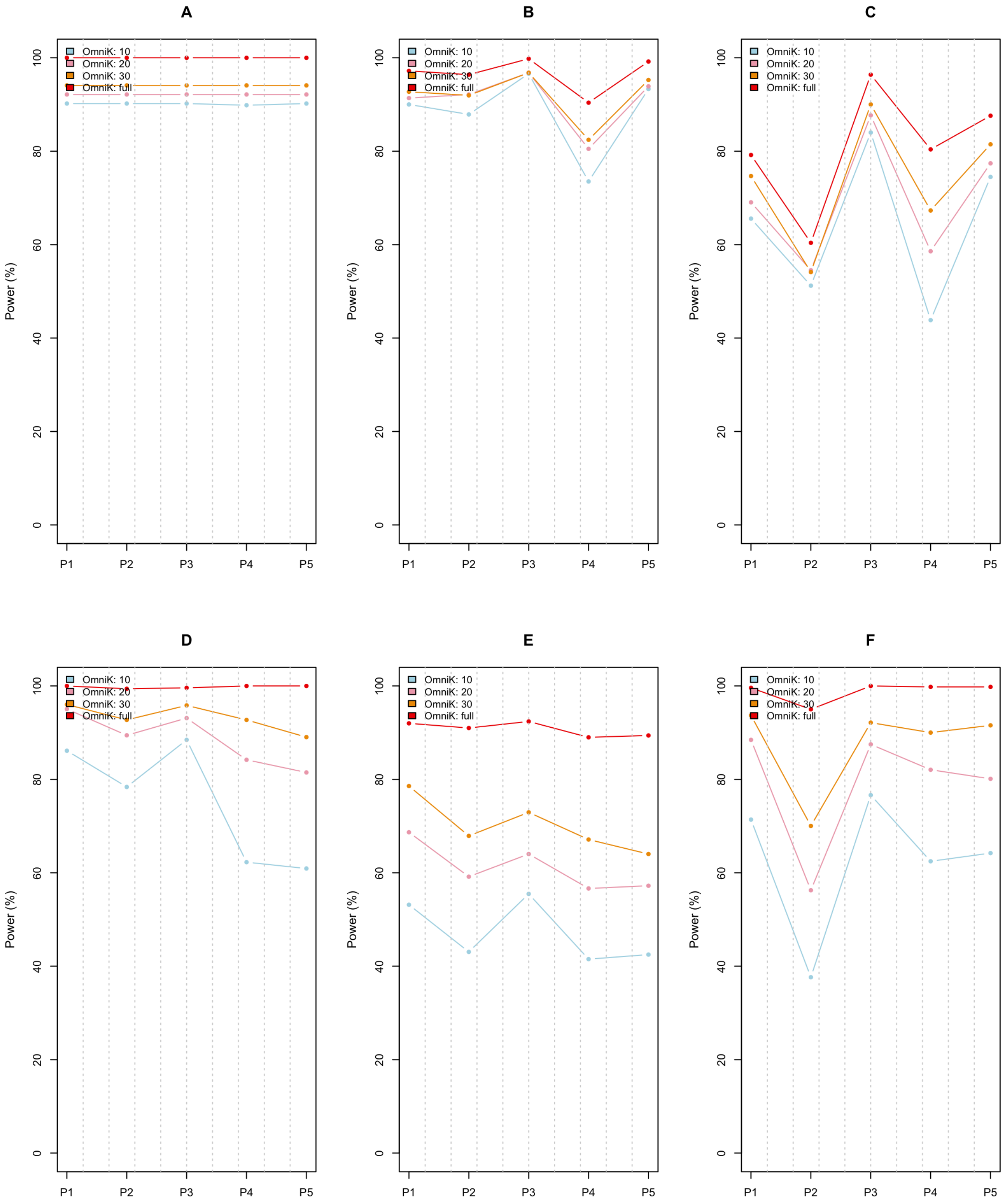

S22 Figure: Empirical powers for continuous response and observational study ($n = 200$) using OmniK with 10 df, OmniK with 20 df, OmniK with 30 df and OmniK with full df. * The parameters of the Dirichlet-multinomial distribution were estimated using the Yanai et al's gut microbiome data. * A is for linear relationship with main effects; B is for linear relationship with interaction effects; C is for linear relationship with both of main and interaction effects; D is for nonlinear discrete relationship with main effects; E is for nonlinear discrete relationship with interaction effects; F is for nonlinear discrete relationship with both of main and interaction effects. * P1-P5 represents a selected phylogenetic cluster.

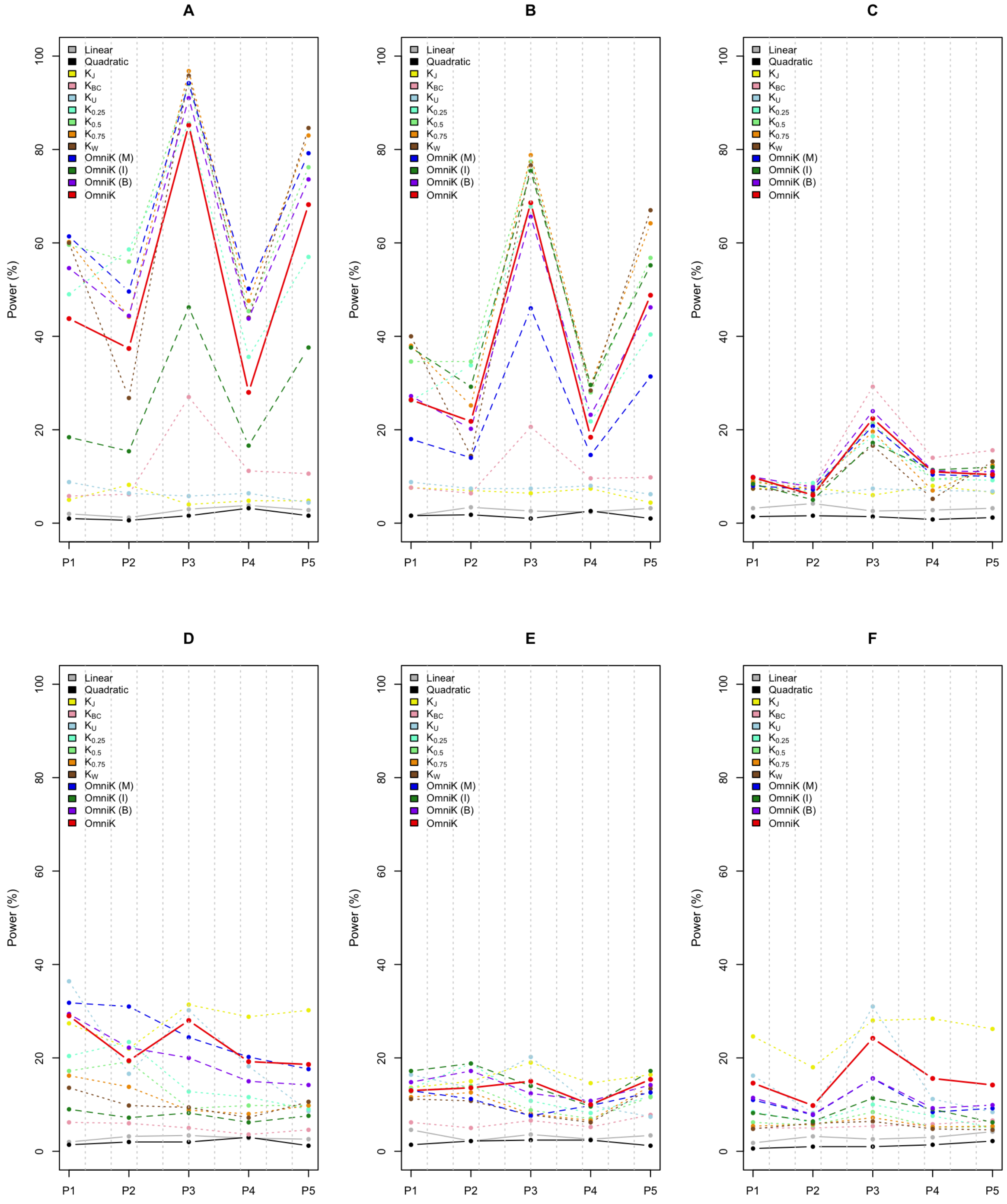

S23 Figure: Empirical powers for binary response and randomized clinical trial ($n = 100$) using (i) existing methods: CKAT based on linear and quadratic kernels, respectively (see Linear and Quadratic); (ii) general kernel machine regression analysis for each ecological kernel (see K_J, K_{BC}, K_U, $K_{0.25}$, $K_{0.5}$, $K_{0.75}$ and K_W); (iii) omnibus testing approach for each endogenous kernel on main effects, interaction effects or both of them (see OmniK (M), OmniK (I), OmniK (B)); and (iv) omnibus testing approach across all endogenous and input kernels (see OmniK). * The parameters of the Dirichlet-multinomial distribution were estimated using the Yanai et al's gut microbiome data.* A is for linear relationship with main effects; B is for linear relationship with interaction effects; C is for linear relationship with both of main and interaction effects; D is for nonlinear discrete relationship with main effects; E is for nonlinear discrete relationship with interaction effects; F is for nonlinear discrete relationship with both of main and interaction effects. * P1-P5 represents a selected phylogenetic cluster.

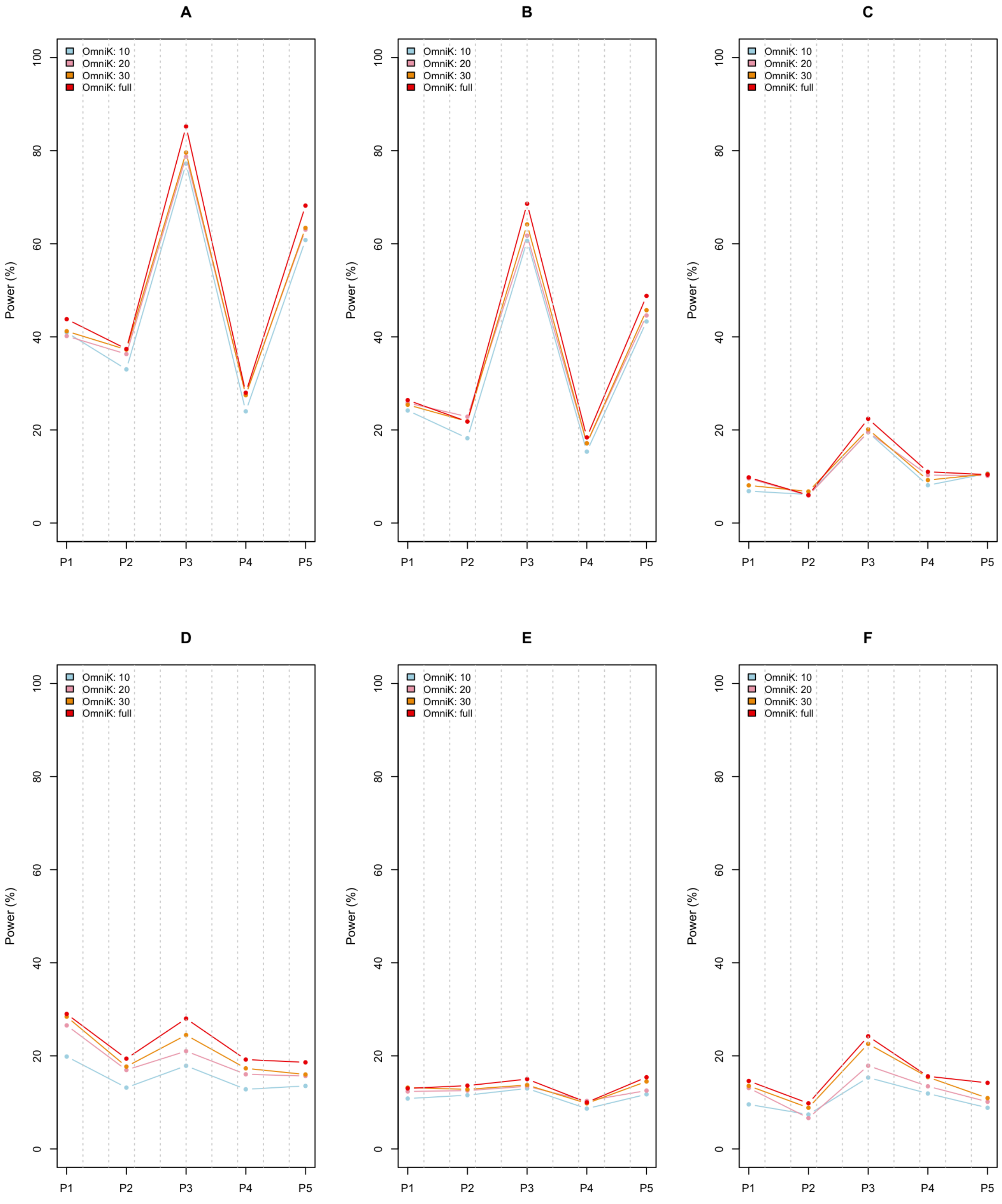

S24 Figure: Empirical powers for binary response and randomized clinical trial ($n = 100$) using OmniK with 10 df, OmniK with 20 df, OmniK with 30 df and OmniK with full df. * The parameters of the Dirichlet-multinomial distribution were estimated using the Yanai et al's gut microbiome data. * A is for linear relationship with main effects; B is for linear relationship with interaction effects; C is for linear relationship with both of main and interaction effects; D is for nonlinear discrete relationship with main effects; E is for nonlinear discrete relationship with interaction effects; F is for nonlinear discrete relationship with both of main and interaction effects. * P1-P5 represents a selected phylogenetic cluster.

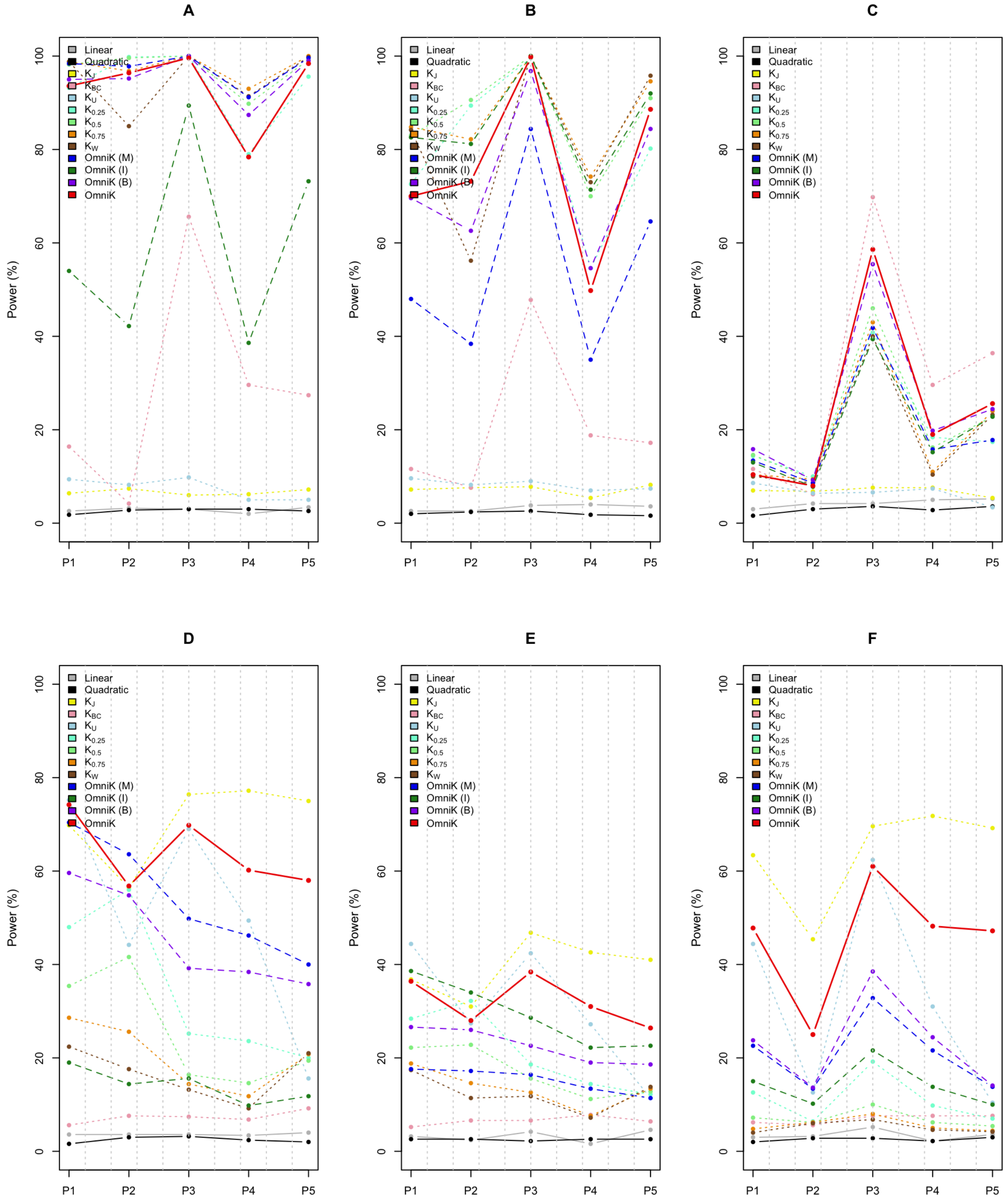

S25 Figure: Empirical powers for binary response and randomized clinical trial ($n = 200$) using (i) existing methods: CKAT based on linear and quadratic kernels, respectively (see Linear and Quadratic); (ii) general kernel machine regression analysis for each ecological kernel (see K_J, K_{BC}, K_U, $K_{0.25}$, $K_{0.5}$, $K_{0.75}$ and K_W); (iii) omnibus testing approach for each endogenous kernel on main effects, interaction effects or both of them (see OmniK (M), OmniK (I), OmniK (B)); and (iv) omnibus testing approach across all endogenous and input kernels (see OmniK). * The parameters of the Dirichlet-multinomial distribution were estimated using the Yanai et al's gut microbiome data.* A is for linear relationship with main effects; B is for linear relationship with interaction effects; C is for linear relationship with both of main and interaction effects; D is for nonlinear discrete relationship with main effects; E is for nonlinear discrete relationship with interaction effects; F is for nonlinear discrete relationship with both of main and interaction effects. * P1-P5 represents a selected phylogenetic cluster.

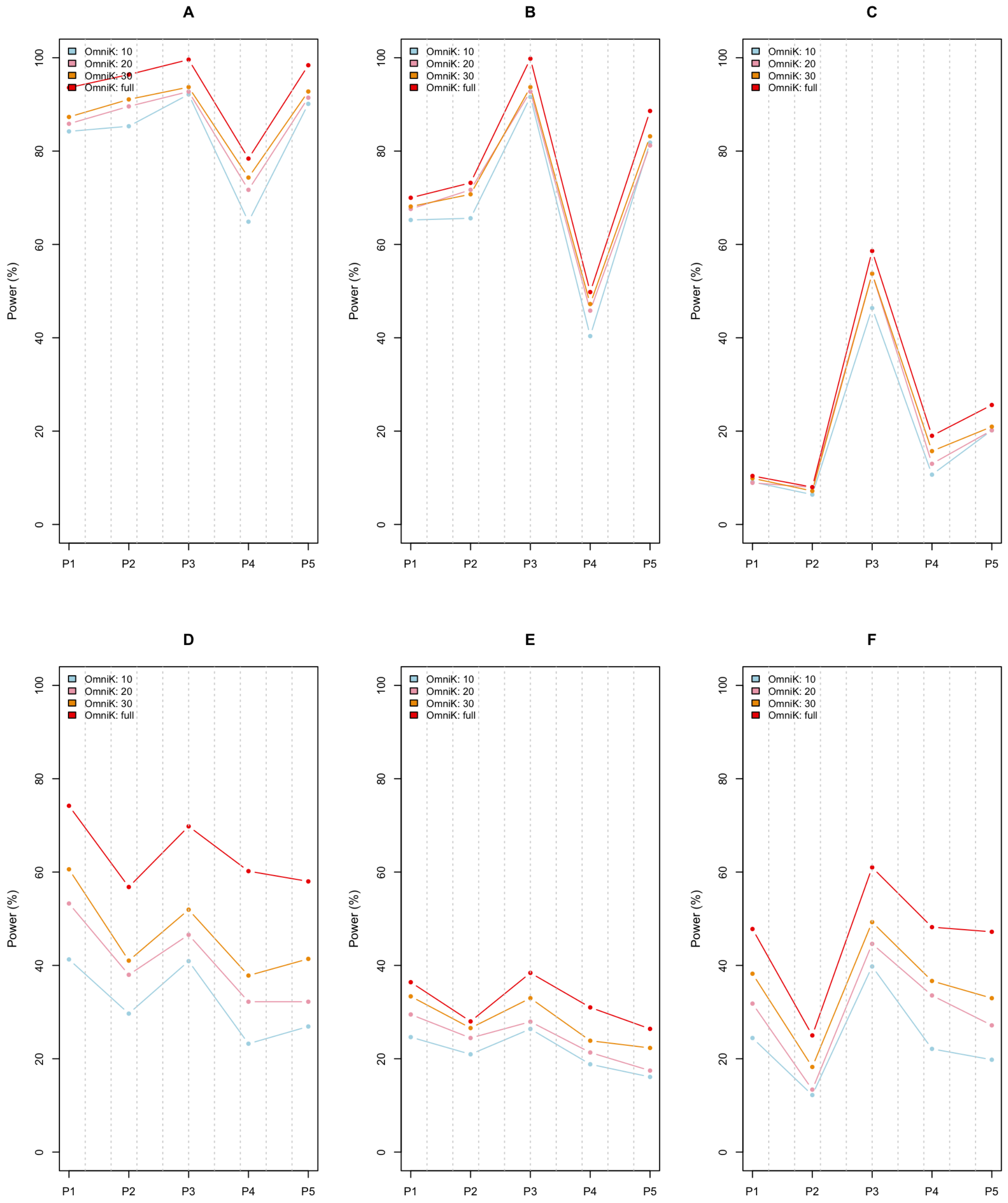

S26 Figure: Empirical powers for binary response and randomized clinical trial ($n = 200$) using OmniK with 10 df, OmniK with 20 df, OmniK with 30 df and OmniK with full df. * The parameters of the Dirichlet-multinomial distribution were estimated using the Yanai et al's gut microbiome data. * A is for linear relationship with main effects; B is for linear relationship with interaction effects; C is for linear relationship with both of main and interaction effects; D is for nonlinear discrete relationship with main effects; E is for nonlinear discrete relationship with interaction effects; F is for nonlinear discrete relationship with both of main and interaction effects. * P1-P5 represents a selected phylogenetic cluster.

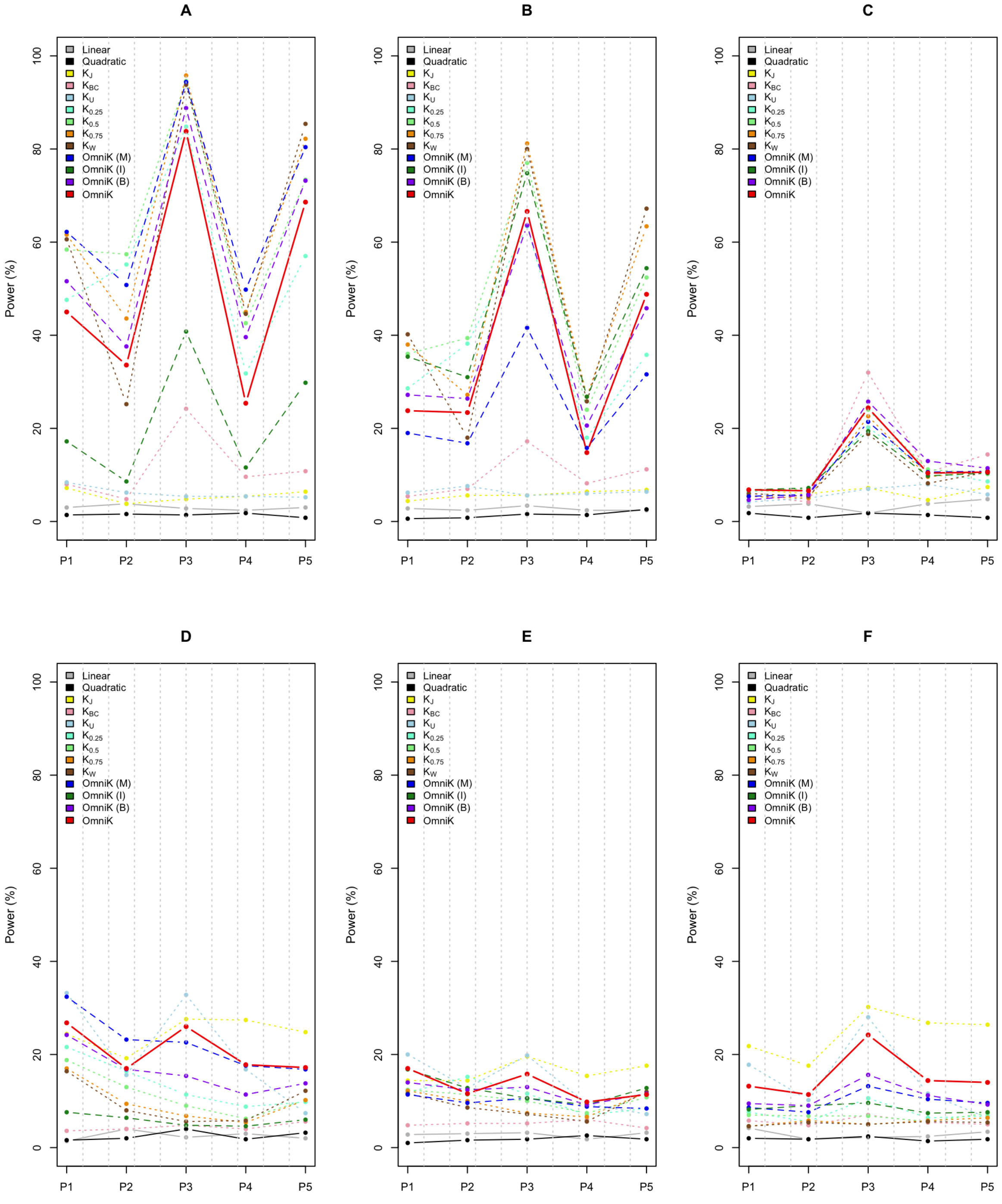

S27 Figure: Empirical powers for binary response and observational study ($n = 100$) using (i) existing methods: CKAT based on linear and quadratic kernels, respectively (see Linear and Quadratic); (ii) general kernel machine regression analysis for each ecological kernel (see K_J, K_{BC}, K_U, $K_{0.25}$, $K_{0.5}$, $K_{0.75}$ and K_W); (iii) omnibus testing approach for each endogenous kernel on main effects, interaction effects or both of them (see OmniK (M), OmniK (I), OmniK (B)); and (iv) omnibus testing approach across all endogenous and input kernels (see OmniK). * The parameters of the Dirichlet-multinomial distribution were estimated using the Yanai et al's gut microbiome data.* A is for linear relationship with main effects; B is for linear relationship with interaction effects; C is for linear relationship with both of main and interaction effects; D is for nonlinear discrete relationship with main effects; E is for nonlinear discrete relationship with interaction effects; F is for nonlinear discrete relationship with both of main and interaction effects. * P1-P5 represents a selected phylogenetic cluster.

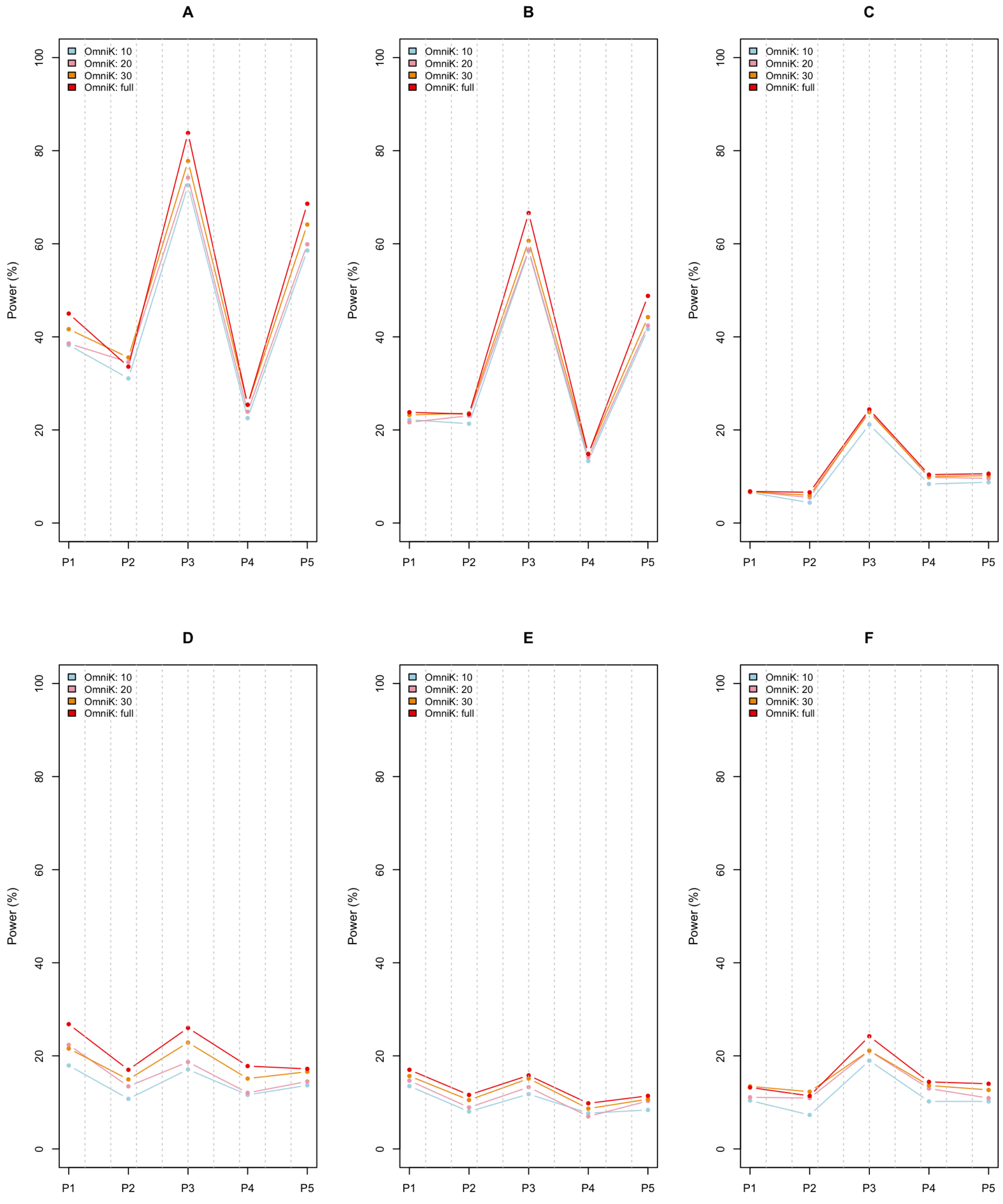

S28 Figure: Empirical powers for binary response and observational study ($n = 100$) using OmniK with 10 df, OmniK with 20 df, OmniK with 30 df and OmniK with full df. * The parameters of the Dirichlet-multinomial distribution were estimated using the Yanai et al's gut microbiome data. * A is for linear relationship with main effects; B is for linear relationship with interaction effects; C is for linear relationship with both of main and interaction effects; D is for nonlinear discrete relationship with main effects; E is for nonlinear discrete relationship with interaction effects; F is for nonlinear discrete relationship with both of main and interaction effects. * P1-P5 represents a selected phylogenetic cluster.

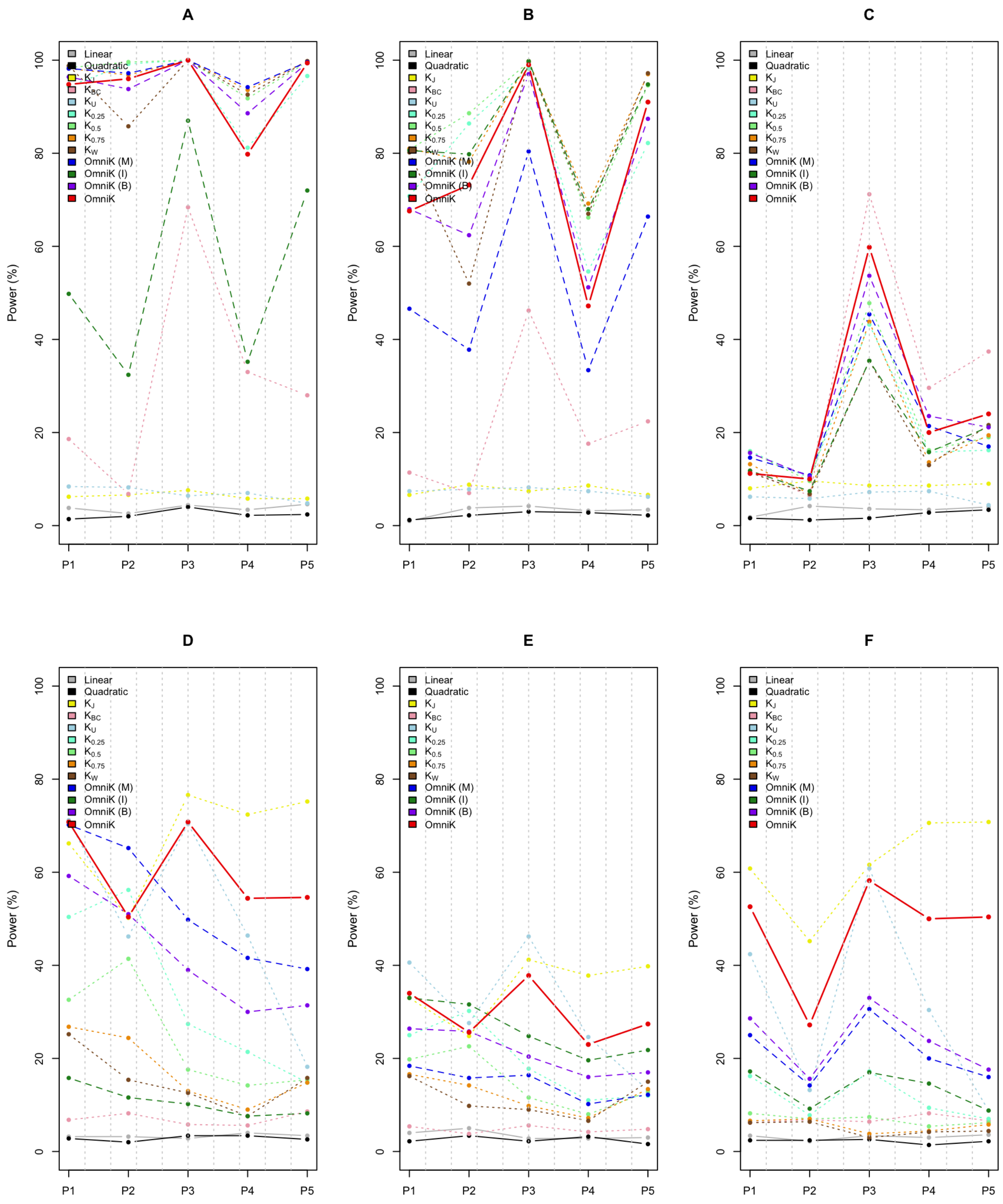

S29 Figure: Empirical powers for binary response and observational study ($n = 200$) using (i) existing methods: CKAT based on linear and quadratic kernels, respectively (see Linear and Quadratic); (ii) general kernel machine regression analysis for each ecological kernel (see K_J, K_{BC}, K_U, $K_{0.25}$, $K_{0.5}$, $K_{0.75}$ and K_W); (iii) omnibus testing approach for each endogenous kernel on main effects, interaction effects or both of them (see OmniK (M), OmniK (I), OmniK (B)); and (iv) omnibus testing approach across all endogenous and input kernels (see OmniK). * The parameters of the Dirichlet-multinomial distribution were estimated using the Yanai et al's gut microbiome data.* A is for linear relationship with main effects; B is for linear relationship with interaction effects; C is for linear relationship with both of main and interaction effects; D is for nonlinear discrete relationship with main effects; E is for nonlinear discrete relationship with interaction effects; F is for nonlinear discrete relationship with both of main and interaction effects. * P1-P5 represents a selected phylogenetic cluster.

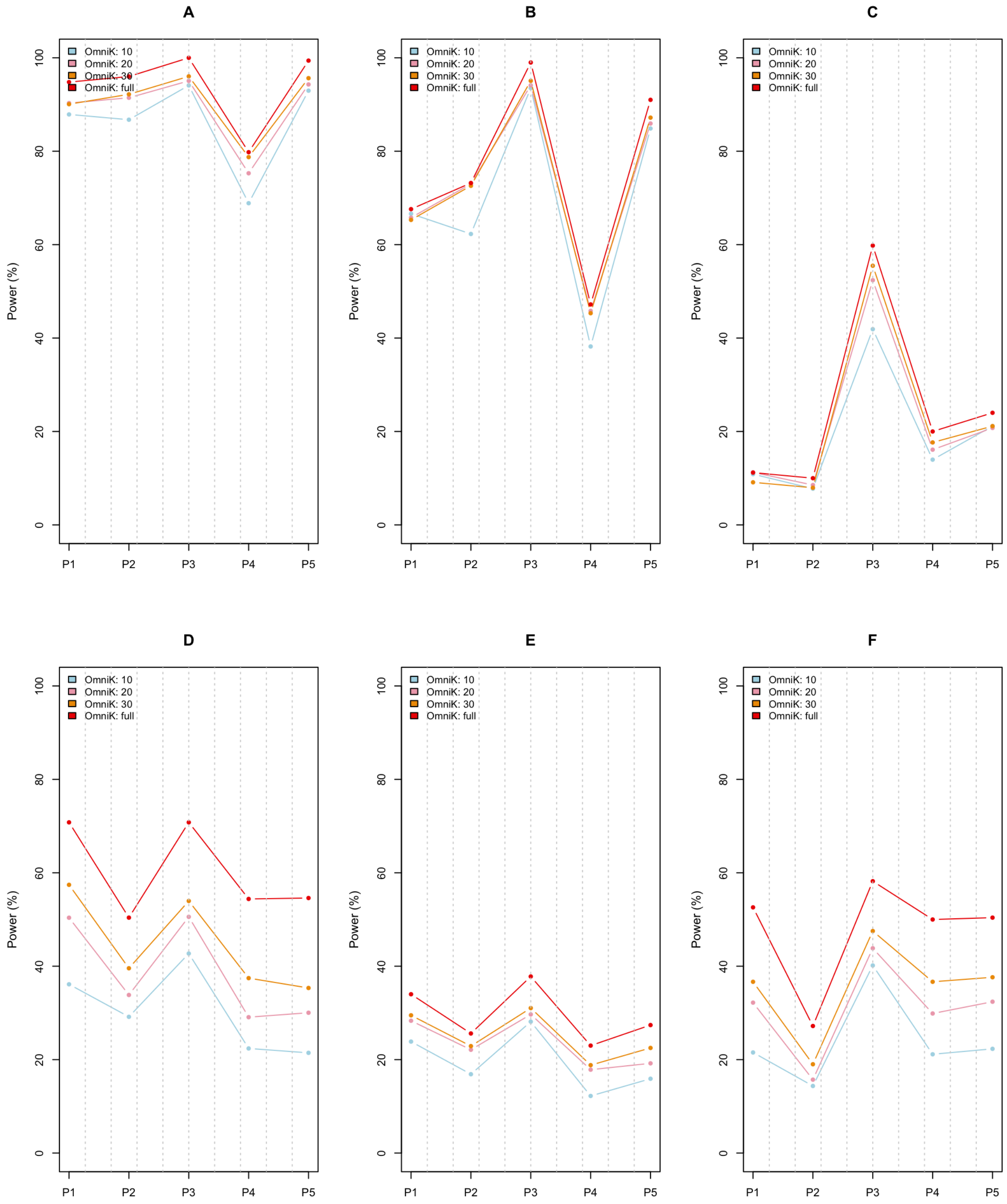

S30 Figure: Empirical powers for binary response and observational study ($n = 200$) using OmniK with 10 df, OmniK with 20 df, OmniK with 30 df and OmniK with full df. * The parameters of the Dirichlet-multinomial distribution were estimated using the Yanai et al's gut microbiome data. * A is for linear relationship with main effects; B is for linear relationship with interaction effects; C is for linear relationship with both of main and interaction effects; D is for nonlinear discrete relationship with main effects; E is for nonlinear discrete relationship with interaction effects; F is for nonlinear discrete relationship with both of main and interaction effects. * P1-P5 represents a selected phylogenetic cluster.
